# Li Dynamics in Mixed Ionic-Electronic Conducting Interlayer of All-Solid-State Li-metal Batteries

**DOI:** 10.1021/acs.nanolett.3c04072

**Published:** 2024-01-25

**Authors:** Daxian Cao, Yuxuan Zhang, Tongtai Ji, Xianhui Zhao, Ercan Cakmak, Soydan Ozcan, Michael Geiwitz, Jean Bilheux, Kang Xu, Ying Wang, Kenneth Stephen Burch, Qingsong Howard Tu, Hongli Zhu

**Affiliations:** † Department of Mechanical and Industrial Engineering, Northeastern University, Boston, Massachusetts 02115, United States; ‡ Neutron Scattering Division, Oak Ridge National Laboratory, Oak Ridge, Tennessee 37831, United States; § Environmental Sciences Division, Oak Ridge National Laboratory, 1 Bethel Valley Road, Oak Ridge, Tennessee 37830, United States; ∥ Materials Science and Technology Division, Oak Ridge National Laboratory, Oak Ridge, Tennessee 37831, United States; ⊥ Manufacturing Science Division, Oak Ridge National Laboratory, 1 Bethel Valley Road, Oak Ridge, Tennessee 37830, United States; # Department of Physics, Boston College, Chestnut Hill, Massachusetts 02467, United States; ∇ Battery Science Branch, Sensor and Electron Devices Directorate, CCDC Army Research Laboratory, Adelphi, Maryland 20783-1197, United States; ◆ Mechanical Engineering, Rochester Institute of Technology, Rochester, New York 14623, United States

**Keywords:** lithium−metal anode, all-solid-state batteries, mixed ionic-electronic conductor (MIEC), *operando* neutron imaging, Raman spectroscopy, mechano-chemo-electrochemical processes

## Abstract

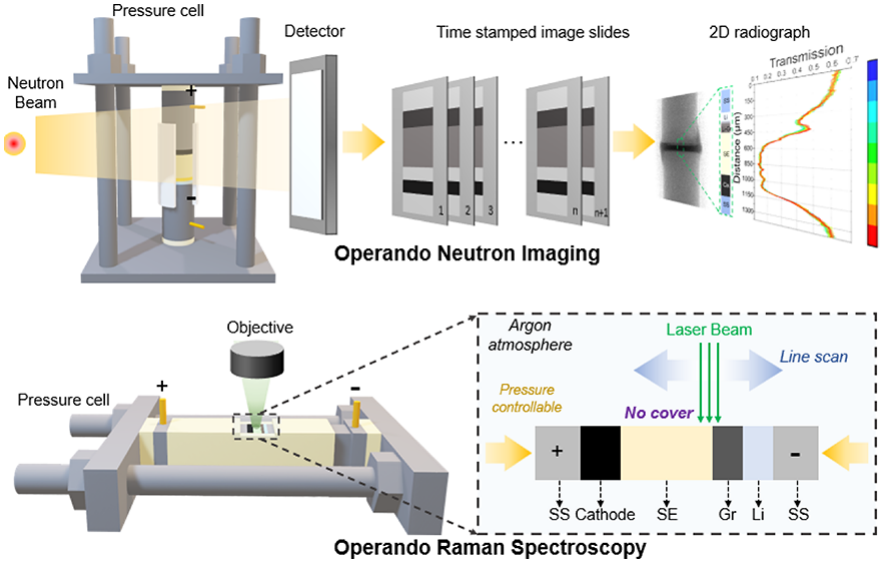

Lithium–metal (Li^0^) anodes potentially enable all-solid-state batteries with high energy density. However, it shows incompatibility with sulfide solid-state electrolytes (SEs). One strategy is introducing an interlayer, generally made of a mixed ionic-electronic conductor (MIEC). Yet, how Li behaves within MIEC remains unknown. Herein, we investigated the Li dynamics in a graphite interlayer, a typical MIEC, by using *operando* neutron imaging and Raman spectroscopy. This study revealed that intercalation-extrusion-dominated mechanochemical reactions during cell assembly transform the graphite into a Li-graphite interlayer consisting of SE, Li^0^, and graphite-intercalation compounds. During charging, Li^+^ preferentially deposited at the Li-graphite|SE interface. Upon further plating, Li^0^-dendrites formed, inducing short circuits and the reverse migration of Li^0^. Modeling indicates the interface has the lowest nucleation barrier, governing lithium transport paths. Our study elucidates intricate mechano-chemo-electrochemical processes in mixed conducting interlayers. The behavior of Li^+^ and Li^0^ in the interlayer is governed by multiple competing factors.

All-solid-state lithium metal batteries (ASLMBs) promise high energy density, safety, and packing density, attracting great interest.^1,2^ Sulfide solid electrolytes (SEs) like Li_10_GeP_2_S_12_,^3^ Li_5.5_PS_4.5_Cl_1.5_,^4^ and Li_9.54_Si_1.74_P_1.44_S_11.7_Cl_0.3_^5^ demonstrate >10 mS/cm room temperature conductivity, enabling high ASLMB performance.^6^ However, issues like poor cycling, low Coulombic efficiency, and rate capability under high cathode mass loading persist due to unfavorable chemistry, electrochemistry, and mechanics between the Li metal (Li^0^) and sulfide SE.^7,8^ Approaches to improve interfacial stability include interlayers,^9^ passivation layers,^10^ electrolyte doping,^11^ and densification,^12^ but extending the lifetime at high areal capacities remains challenging.

Introducing an interlayer between SE and Li^0^ is one of the most adopted strategies.^9,13−16^ Yet, some reported interlayers, like the silver–carbon composite^13^ and graphite,^14,15^ are typical mixed ionic-electronic conductors (MIEC).^17^ Graphite was studied here as a representative MIEC interlayer to gain insights into Li dynamics that are likely to extend to other mixed conductors. The graphite interlayer can enable a Li^0^/SE/Li^0^ symmetric cell to operate at a remarkable critical current density (CCD) of 10 mA cm^–2^ (Figure S1), seemingly suggesting that electron insulation is optional. Furthermore, full cells with different graphite interlayer thicknesses were assembled and tested at a rate of C/20 with a cathode mass loading of 20 mg cm^–2^. However, “soft short” happened at the fifth and fourth cycles individually (Figure S2), and the result is independent of the interlayer thickness. Therefore, a deep understanding of the MIEC interlayer is desired for practical application.

The behavior of Li^0^ and Li^+^ in the MIEC interlayer follows more complex mechano-chemistry and mechano-electrochemistry, which are closely entangled (Figure 1a). Since stacking pressure in megapascals is generally applied during cell assembly to obtain an intimate contact, the MIEC experiences a mechanochemical reaction with Li^0^ upon battery construction. Due to its soft and ductile nature (the yield strength of Li^0^ is 0.8 MPa), Li^0^ can be extruded into the MIEC through cracks, voids, and defects (Protocol 1). At the same time, Li^0^ can react with MIEC through intercalation (such as graphite) or alloying (such as silver, silicon, and aluminum) (Protocol 2), which is accelerated by the pressure. The thermodynamic states of both MIEC and Li^0^ affect the following electrochemistry result. For summary, the MIECs could respond to Li^+^ in three different pathways depending on the electrochemical potential it is subject to (1) reacting with Li^+^ through intercalation (forming graphite-intercalation-compounds (GICs)) or alloying (forming Li-silver, Li-silicon, and Li-aluminum alloys in silver, silicon, and aluminum, respectively) (Path 1); (2) Li^+^ transport through the MIEC and deposit onto the preexisting Li^0^ (Path 2); and (3) MIEC transport electron from Li^0^ and reduce the Li^+^, thus inducing Li^0^-plating at the MIEC|SE interface (Path 3). The performance of batteries relies on the competition of these three paths, and path 2 is the favored path for a stable interface for long cycling life.

**Figure 1:**
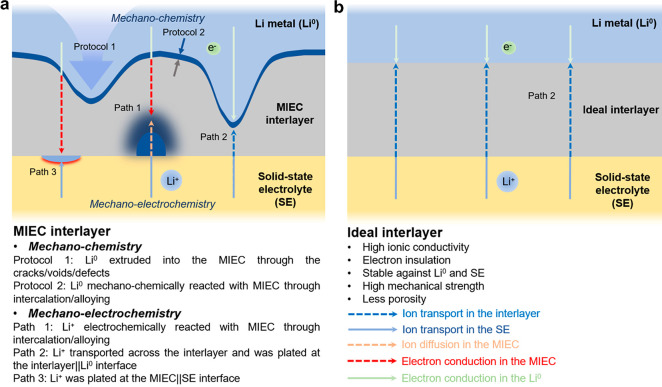
Li^0^ and Li^+^ evolution at the interlayers in ASLMBs. Schematic of the Li^0^ and Li^+^ behavior at the anode side in ASLMBs using interlayer (a) made of mixed ionic-electronic conductor (MIEC); (b) with only ionic conductivity during the plating process.

To probe these mechanisms, we designed a graphite interlayer ASLMB for *operando* neutron imaging and Raman spectroscopy. These nondestructive techniques can uniquely elucidate Li evolution during operation. The cell comprised a single-crystal LiNi_0.8_Mn_0.1_Co_0.1_O_2_ (NMC) cathode, Li_5.4_PS_4.4_Cl_1.6_ solid electrolyte, 100 μm Li anode, and a 25–30 μm graphite interlayer between the Li and electrolyte (Note S1). Driven by the intercalation-extrusion nature of Li^0^, the graphite layer underwent a mechanochemical reaction with Li^0^ during stacking, resulting in the formation of the Li-graphite layer with a complex composition consisting of Li^0^, SE powder, and diluted GICs. Given this unique structure, in the full cell test, the preferential deposition of Li^0^ at the interface between Li-graphite and the SE layer was first observed, followed by Li^0^ deposition in the Li-graphite. However, no intercalation occurred in the Li-graphite. This Li^+^ evolution is determined by the lowest overpotential of nucleation at the Li-graphite and SE layer interface, compared with the higher energy barriers associated with the intercalation of Li^+^ in graphite and the Li^+^ transport through the Li-graphite to Li^0^. Eventually, the plated Li^0^ penetrated the SE, inducing the short circuit of the ASLMB and the subsequent reversed Li^0^ transfer from the anode to the cathode during charge.

For the first time, this work well explained the complex dynamics of Li^0^ and Li^+^ in MIEC interlayer results from combined mechanochemical and mechano-electrochemical reactions and resulted in three competitive paths. From this study, we concluded that this interlayer should meet the criteria of high ionic conductivity, electron insulation, stability against Li^0^ and SE, high mechanical strength, and low porosity (Figure 1b). In particular, electron insulation is regarded as critical in avoiding Li^0^ deposition at the interlayer|SE interface. These insights provide guiding principles to engineer interfaces for stable cycling.

## Mechano-Chemistry Reaction of Li-Graphite before Electrochemical Cycling

**Figure 2:**
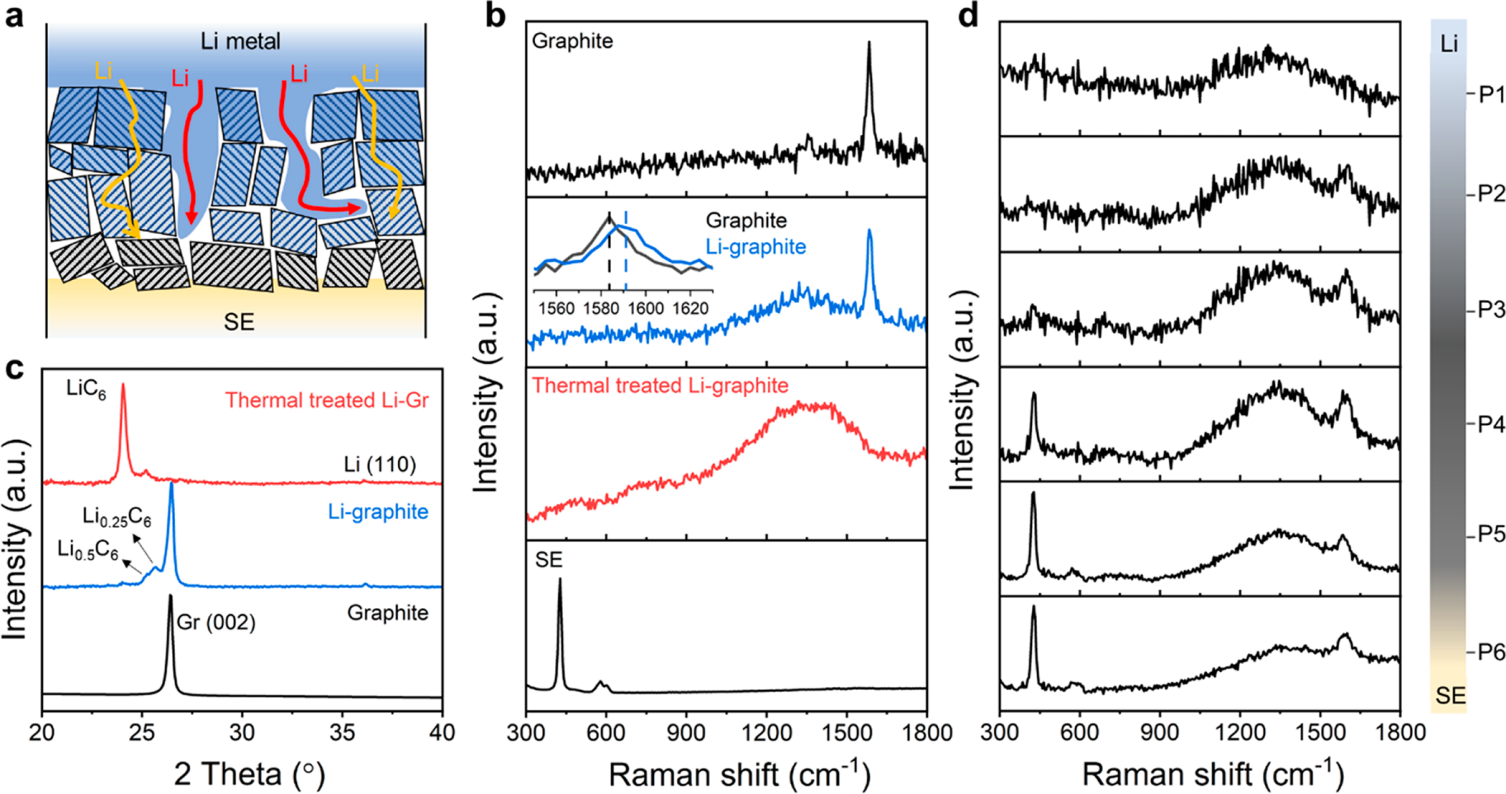
Mechano-chemistry investigation of Li-graphite interlayer. (a) Schematics of the two different mechanochemical protocols. (b) Raman spectra of pristine graphite, pristine Li-graphite, thermally treated Li-graphite, and SE. (c) XRD patterns of Li-graphite, thermally treated Li-graphite, and pristine graphite. (d) Raman spectra of Li-graphite at different positions along the cross section. The side schematic in panel d shows the positions along the cross section of the Li-graphite.

A high pressure of 300 MPa was applied to the cell stacking to achieve intimate contact, resulting in a low interface resistance. As reported, the overpotential in the cell operated under 100 MPa was only 63% of that under a pressure of 3 MPa.^14^ However, the graphite interlayer under stacking pressure changed into complicated compositions. Li^+^ can intercalate into the 2D structured graphite (yellow path) and be extruded through the tortuous pores in the graphite layer (red path) in a metallic form (Figure 2a). This initial mechano-chemistry reaction result controls the Li^+^ behavior in the following electrochemical reactions.

Driven by thermodynamics and accelerated by the applied pressure, Li^0^ intercalation occurs in the Li-graphite layer. Raman spectra show evidence of the formation of GICs^18^ accompanied by increased disorder (Figure 2b, Note S2); however, the Li-graphite maintains the same black color as the pristine graphite^19^ (Figure S3). The morphology of the smooth graphite sheets becomes rough and plicate after the reaction (Figure S4). X-ray diffraction (XRD) reveals the existence of the high-stage intercalated GICs (Figure 2c), mainly Li_0.25_C_6_, Li_0.5_C_6_, a minor presence of LiC_6_, and the dominant graphite. Since the intercalation initializes from the graphite edges, the GIC is likely graphite that only contains intercalation at the edge sites while the interior maintains the graphitic structure. Upon further heating at 160 °C for 12 h, the Li-graphite becomes golden (Figure S5), suggesting the formation of the stage-I GIC,^19^ LiC_6_. However, the Raman spectrum shows a broad feature likely resulting from electronic Raman of highly intercalated graphite.^18,20−23^ There is no clear G band in comparison to LiC_6_ prepared through electrochemical intercalation (Figure S6). The absence of splitting and G band indicates the GIC is still close to Stage I. These results further indicate that graphite and Li^0^ undergo a heterogeneous mechanochemical reaction resulting in highly disordered GIC.

To further investigate the compositions across the Li-graphite layer, the Li^0^|Li-graphite|SE cross-section was divided into six distinct regions (designated as P1 on the Li^0^ side through P6 on the SE side) for Raman spectra collection (Figure 2d). When scanning from P6 to P1, the G peak intensity gradually decreased (Table S1), accompanied by peak shifts to higher wavenumber compared with those of the pure graphite (Figure S7a). These blue shifts of the G peak represent the Li^+^ intercalation, and the intercalation amount increases with the layer closer to the Li^0^. The reduced G-peak intensities reflect the reduction in the optical skin depth caused by the enhanced electronic conductivities of the GICs as the intercalation proceeds.^21^ The intensity ratio of the broad feature centered at 1350 cm^–1^ to the G peak increased from 1.19 to 3.42, in contrast to that of 0.14 in pure graphite. This confirms the correlation between the disorder and the presence of Li^0^. Therefore, the anode behavior was successfully tracked by monitoring the peak intensity and shift changes of the broad feature at ∼1350 cm^–1^ and G peaks in the Raman spectra, respectively. At P4, P5, and P6, the peaks representing SE can be well-defined (Note S3). Furthermore, since the cold-pressed SE inevitably contained some cavities (Figure S8), the Li-graphite could be compressed underneath the SE surface under high stacking pressure. Consequently, the Li-graphite interlayer transformed into a complex mixture of Li^0^, diluted GICs, and SE.

## *Operando* Neutron Imaging Investigation of Mechano-Electrochemistry

**Figure 3:**
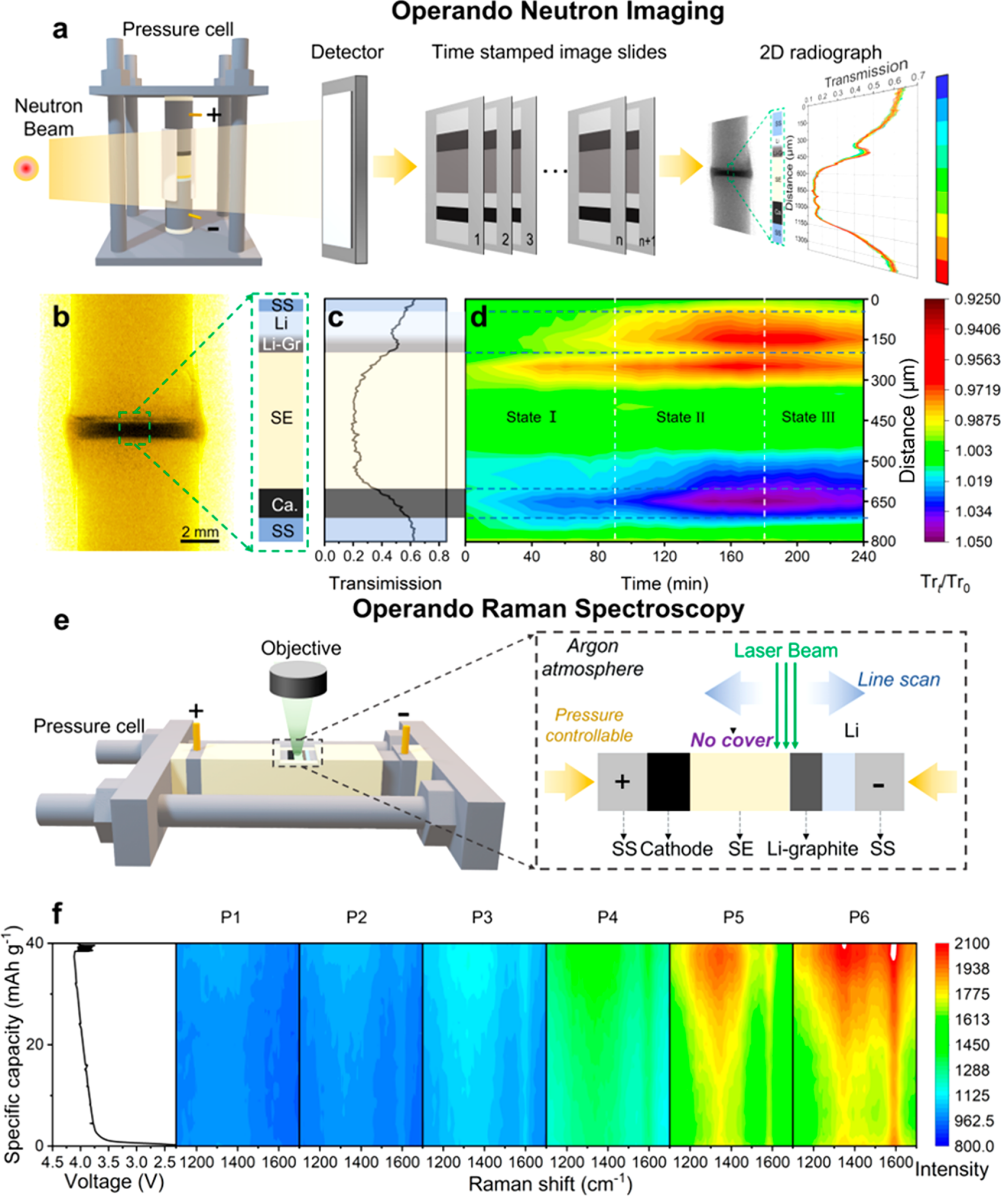
*Operando* neutron imaging and Raman spectroscopy investigation of the Li evolution in the ASLMBs. (a) Schematic of the *operando* neutron imaging. (b) Normalized neutron radiography image of the ASLMB to identify each component based on the neutron attenuation differentiation. The inset schematic displays the cell configuration. (c) Quantified neutron transmission in the labeled region along the cross section of the ASLMB. The inset schematic assigns the neutron transmission to different components. (d) Dynamic transmission evolution during charging. The green, warm, and cold colors represent no obvious changes, enriched Li, and Li depletion, respectively, compared to the pristine state. (e) Schematic of *operando* Raman spectroscopy. (f) Intensity mappings of Raman spectra in the range of 1100–1700 cm^–1^ as a function of charging time. The figure at the left end displays the galvanostatic charge profile of the ASLMB.

In this work, neutron imaging, which has a high sensitivity to Li (either Li^0^ or Li^+^) and high penetrating ability through the cell wall,^24^ was used to track the Li distribution *in operando* in the ASLMB (Figure 3a, Note S4). A normalization treatment (Note S5) was applied to the raw neutron images (Figure S9) to enhance the signal-to-noise ratio. As a result, 2D neutron radiography (Figure 3b) effectively characterized the laminated structure of the ASLMB (Note S6). Furthermore, we plotted the neutron transmission data across the ASLMB (Figure 3c) (Note S7) to identify different component positions. The interfaces among adjacent layers, including the SE|Li-graphite layer and the SE|cathode interfaces, were well-defined through the derivative transmission (Figure S10).

The ASLMB is normally charged at 0.2 mA cm^–2^ for 3 h with a specific capacity of 30 mAh g^–1^, and then the voltage gradually drops, indicating the occurrence of a “short circuit”^25^ (Figure S11). Since the transmission change is imperceptible in the image (Figure S12), a further normalization treatment was applied to amplify the changes. The change in neutron transmission (Tr_*t*_) at the charging time (*t*) was evaluated by comparing the transmission change ratio (Tr_*t*_/Tr_0_) with the initial transmission (Tr_0_) (Note S8). Figure S13 presents a series of time-stamped neutron radiography images normalized to the initial state. The enhanced dark and bright regions represent enriched and depleted Li, respectively. The inset schematic shows the cell configuration. Prior to battery failure (0–180 min), Li^+^ departs from the cathode side and accumulates at the anode. After the short circuit (180–240 min), Li gradually loses from the anode, and the Li in the cathode increases. The neutron imaging provides a real-time visualization of the Li evolution and how the ASLMB behaves once a short circuit occurs (Video S1). To the best of our knowledge, this is the first time the Li evolution in MIEC under mechano-electrochemistry in ASLMB has been visualized in *operando* mode.

The mapping derived from the quantified Tr_*t*_/Tr_0_ provides further details (Figure 3d). The warm color (Tr_*t*_/Tr_0_ < 1) represents the enrichment of Li; the cold color (Tr_*t*_/Tr_0_ > 1) indicates the depletion of Li; and the green (Tr_*t*_/Tr_0_ close to 1) indicates no obvious change. Overall, the cathode side shows evidence of Li depletion during the test, whereas the anode side shows a buildup of Li. More specifically, three stages occur during the charging process. At the beginning (0–90 min, State I), the concentration of Li mainly increases at the Li-graphite|SE interface. Because Li-graphite is partially squeezed into the SE layer and the surface voids can host the plated Li^0^, it shows the Li enrichment is mainly located on the SE side. In comparison, the depletion of Li occurs homogeneously in the cathode, whereas the part extruded into the SE shows a relatively weak intensity because of the diluted concentration. At the second stage (90–180 min, state II), Li concentrates at the Li-graphite|SE interface, the Li-graphite, as well as in the Li^0^ regions, proving the Li^+^ could transport across the Li-graphite layer. After 180 min of charge (state III), the ASLMB is short-circuited. Though the battery is still charging, the cell shows an inverse Li concentration trend: the anode loses Li and the cathode gains Li. However, it is difficult to detect the dendrite in the SE because of the low dimension of the dendrite and its limited contrast with the SE. Another *operando* test with the same setup confirms our observation that the Li accumulates at the interface first and then beneath the Li-Gr (Figure S14 and Note S9).

## *Operando* Raman Spectroscopy Investigation of Li-Graphite Evolution

*Operando* Raman, widely used to characterize graphite with high spatial resolution, was complimented with neutrons to indirectly evaluate the Li^+^ behaviors, i.e., intercalation versus plating in MIEC, as neutron imaging alone is incapable of distinguishing between the Li^0^ and Li^+^ (Figure 3e, Note S10). A line scan was performed to collect the real-time Raman spectra from positions P1 to P6 during the battery test. Under a current density of 0.2 mA cm^–2^, the ASLMB exhibited an unstable potential after being normally charged for 230 min (equaling to a specific capacity of 38 mAh g^–1^), suggesting that a “short circuit” occurred (Figure 3f). The Raman intensity mapping shows the intensity evolution with time with the relatively warmer and colder colors representing the intensity enhancement and attenuation, respectively. Overall, the Raman spectra at all of the positions showed intensity enhancements as the battery charged.

In further detail (Figure S15), we observed no significant change in the G-peak position or evidence for splitting consistent with the intercalation stage being unchanged. At positions P1, P2, and P3 we observed an overall enhancement of the Raman spectra with no specific region being enhanced. Regions P4, P5, and P6 displayed similar enhancements; however, the broad feature from the electronic Raman is more strongly enhanced than that of the G-peak or the overall background intensity. This is shown via the intensity at specific wavenumber regions (Figure S16). Since an accurate model of the electronic Raman is not available, we focus on the broad feature and G-peak regions, which we fit with overlapping Lorentzians (Figure S17). While these fits can be used to quantitatively determine the change in the material, they qualitatively confirm that the broad feature gains intensity faster than the G-peak. They similarly show the absence of the G-peak. These findings are consistent with previous works^22,23^ showing enhanced signal of the broad feature upon reducing the graphite crystallite size, likely here from the formation of disorder or defects as the Li metal enters.

In particular, the peak intensity remained constant for the initial 60 min (equaling to a specific capacity of 20 mAh g^–1^) and then gradually increased, which agreed with the multistep reaction processes revealed by the neutron imaging. As previously mentioned, the disorder in Li-graphite is related to Li^0^ plating in the interlayer. These findings demonstrate that Li^0^ plating, not Li^+^ intercalation, is the predominant reaction in Li-graphite during charging. Although the GICs were not fully intercalated, Li^+^ was preferentially plated onto rather than intercalated into the Li-graphite. Such a coexistence of Li^0^ and a diluted stage of Li^+^-GIC has never been directly observed in ASLMBs.

The following failure of the ASLMBs is highly related to the Li behavior.^26,27^ The preferred plating of Li^0^ onto the graphite causes direct contact of Li^0^ and SE. Then Li metal above the interlayer propagates and finally penetrates the SE resulting in the short circuit (Figure S18 and Note S11). The origin of this phenomenon is the Li metal deposits above the Li-graphite interlayer. Therefore, the key to a successful MIEC interlayer is to regulate the Li^0^ deposition beneath the MIEC.

## Mechanism of Mechano-Chemistry and Mechano-Electrochemistry

**Figure 4:**
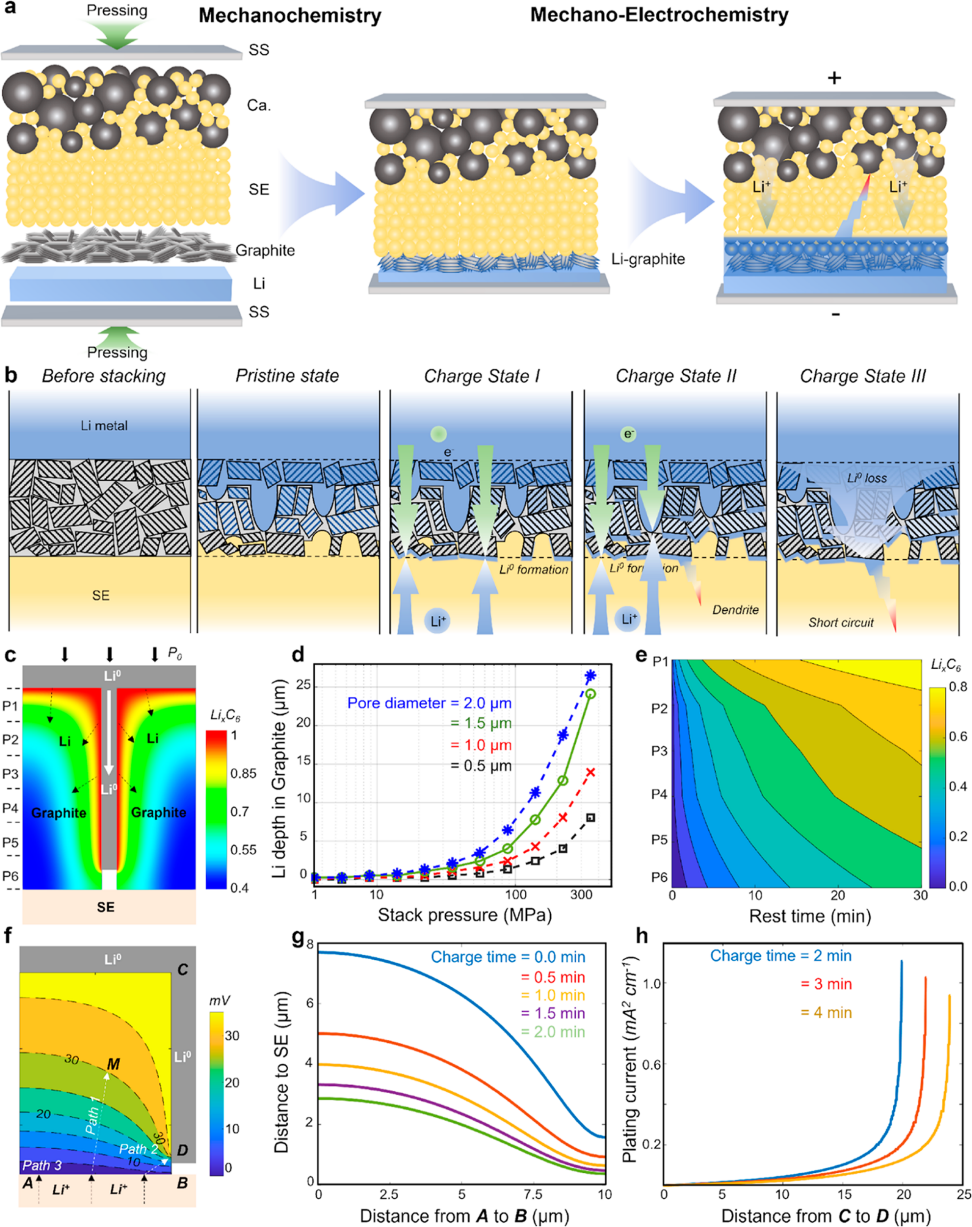
Li evolution within Li-graphite interlayer during the mechano-chemo-electrochemical reaction. (a) Schematic of the mechano-chemistry and mechano-electrochemistry in the ASLMB. (b) Schematic of the ASLMB at different stages: before stacking, pristine state, stage I, stage II, and stage III. (c) Li transport inside the graphite interlayer due to Li^0^ extrusion and intercalation. (d) Depth of extruded Li^0^ at different pore sizes and different stack pressures for 3 min. (e) Temporal evolution of the average Li^+^ content of Li_*x*_C_6_ in different sections. (f) Overpotential needed for Li^+^ to transport inside the Li-graphite layer. (g) Level sets when the overpotential equals the nucleation energy (20 mV) in the first 2 min of cell charging. (h) Deposition current density on the pore surface (C–D) at different charging times.

Figure 4 qualitatively illustrates the Li dynamics in the MIEC interlayer (graphite) from our characterizations. Under stacking pressure, the initial graphite interlayer transforms into a composite interlayer consisting of Li^0^, diluted GICs, and SE particles (pristine state). When charging begins, Li^+^ preferentially plates at the SE/interlayer interface (charge state I) and then deposits inside the Li-graphite interlayer as Li^0^ (charge state II). Finally, the accumulated Li^0^ forms dendrites, causing a short circuit, accompanied by Li^0^ inventory loss at the anode side (charge state III).

An electro-chemo-mechanical model (Note S12) is used to quantitatively analyze the entire process (Figure 4c–h and Video S2). The contour plot in Figure 4c shows the Li^+^ content (*x* in Li_*x*_C_6_) after the cell was mechanically pressed before electrochemical tests. The Li^0^ is extruded deeply into the Li-graphite layer through internal pores (white area), generating more contact surface for Li intercalating into the graphite layer but still maintaining its elemental state, despite the graphite being still not fully lithiated. Figure 4d shows that the Li^0^ extrusion depth is affected by the pore sizes and the applied pressure. The Li^0^ extrusion depth is negligibly small (<3 μm) if the applied stack pressure is less than 100 MPa, but it surges up dramatically under higher pressures. With an average pore size of ∼1.5 μm and a typical tortuosity value (Figure S19) in the dense graphite layer, the Li^0^ can be extruded to a depth comparable to the thickness of the graphite layer (∼25 μm).

According to reports,^28−30^ the Li^+^ diffusivity in the graphite decreases dramatically with increasing Li^+^ content, ranging from ∼10^–9^ cm^2^ s^–1^ (Li_0.1_C_6_) to ∼10^–11^ cm^2^ s^–1^ (LiC_6_). This dynamic evolution of Li^+^ diffusivity, coupled with Li^0^ extrusion (Figure 4d), causes a much more complicated mechanism for Li^+^ transport inside the graphite layer. Figure 4e shows the temporal evolution of the average Li^+^ content (x in Li_*x*_C_6_) of all six regions (P1–P6). Due to Li^0^ extrusion, the value is much larger in all six regions than in the case without Li^0^ extrusion (no pressure, Figure S20). Notably, Li^+^ reaches an average content of Li_0.4_C_6_ in P6, which is consistent with both our XRD results in Figure 2c and the Raman spectra at P6 in Figure 2d.

This composite Li-graphite layer in Figure 4c serves as the initial structure for the cell charging simulation (Figure 4f, representing half of Figure 4c). Unlike the conventional Li^+^ intercalation into graphite layers during charging, Li^0^ plating is more energetically favorable to fill in pores in the Li-graphite layer due to the unique Li distribution. This phenomenon occurs because these pores are close to the graphite|SE interface (within 1–5 μm) and a small overpotential is needed for Li^+^ mass transport. Furthermore, no energy compensation is needed for Li^0^ nucleation (*E*_*n*_ ≈ 20 meV)^30,31^ because Li^0^ is already present at these locations. As indicated in Figure 4f, three paths may exist for the motion of Li^+^ and Li^0^ inside the Li-graphite interlayer during charging: (1) Li^+^ migrates a certain distance within the Li-graphite interlayer and intercalates into graphite layers (such as location M). (2) Li^+^ travels across the MIEC Li-graphite interlayer and plates in the pores where Li^0^ pre-existed (such as location D). (3) Li^+^ is reduced and nucleated as Li^0^ at the Li-graphite|SE interface (A–B line). The actual transport path is determined by the total overpotential needed to complete the path, which is the summation of the Li^+^ transport potential, nucleation barrier (*E*_*n*_ ≈ 20 mV),^31,32^ and the Li^0^ charge-transfer overpotential (η ≈ *R*_charge–transfer-resistance_*i*_applied-current_ < 1 mV, ignored in the current analysis).

The contour in Figure 4f shows the transport potential (in voltage units) required for Li^+^ to migrate within the MIEC Li-graphite interlayer. For example, 30 mV is needed for Li^+^ to move to location M, 15 mV is needed to reach location D, while zero is needed for location B. Therefore, the total overpotential needed for Li^+^ following path 1 (intercalation at location M) is ∼30 mV due to zero nucleation, which is ∼20 mV for path 3 (nucleation at interface point B) due to the Li^0^ nucleation barrier. However, only 15 mV of total overpotential is needed for Li^+^ following path 2 (deposition at location D) due to the zero nucleation barrier. Therefore, Li^+^ prefers to be deposited at location D instead of at location M or B. Each curve in Figure 4g represents the locations in the Li-graphite interlayer where the transport potential equals 20 mV at the respective charging time. Due to the comparable value of total potential for Li^+^ intercalation at these locations (path 1) and Li^0^ deposition at location D (path 3), the preference for Li^+^ staying at these locations at specific charge time is the same as that at location D. Conversely, Li^+^ intercalation is preferred within the region below the curve than at location D; otherwise, Li^0^ deposition at location D is preferred. As the charge time increases, the thickness from the SE|Li-graphite into the interlayer for Li^+^ intercalation dramatically decreases to less than 3um within 2 min, making Li^0^ plating at location D the most preferable path. Figure 4h shows the temporal evolution of the plating current from location C to D, which is proportional to the amount of deposited Li^0^ along interface CD at a specific charge time. For each plating curve, the current increases from C to D due to the concentration effect of the tip point D. The plating curve evolves wider to the positive *x*-direction as the loner charging time, indicating that Li^0^ grows toward location B because of the continuous plating at location D. This trend agrees well with our neutron imaging data in Figure 3c that higher Li concentration is observed on top of the Li-graphite|SE interface and then gradually propagates toward the interlayer.

## Conclusions

In summary, the Li^0^ anode potentially provides an exceedingly high energy density for ASLMB, but its stability with SE, especially sulfide SE, should be further improved. In this work, we investigated Li’s mechanochemical and mechano-electrochemical behavior in MIEC interlayer, with graphite as an example studying case, using *operando* neutron imaging and *operando* Raman spectroscopy. The MIEC interlayer was first divided into six spatial zones with 5 μm for *operando* Raman spectroscopy studies. Under high stacking pressure, the graphite interlayer transformed into a complex Li-graphite interlayer composed of Li^0^, graphite intercalation compounds, and SE, and the chemistry of this Li-graphite interlayer determined the subsequent mechano-electrochemical reaction.

During initial battery charging, the behavior of Li^+^ at the interlayer resulted from three competing dynamics: Li^+^ transportation, intercalation, and deposition. Our results clearly indicate the preferential deposition of Li first at the Li-graphite|SE interface and then within the Li-graphite interlayer. This study reveals that an ideal interlayer should meet key criteria like high ionic conductivity and electron insulation to prevent interfacial plating. Contrary to the view that interlayers should completely block electronic conduction, we propose the MIEC can also work if a low nucleation barrier exists on the Li^0^ metal side to drive Li^+^ transport across the MIEC and deposit on the Li^0^ side. In summary, our visualization highlights the complex mechano-chemo-electrochemical interactions within mixed conducting interlayers, specifically in a graphite interlayer. This interplay, influenced by the amount of plated Li^0^, Li^+^ nucleation barriers, and SE micropore structures, dictates Li evolution and reflects an interwoven relationship between pressure, chemistry, and structural dynamics at the interface.

## Supporting Information Available

Video S1: neutron imaging providing a real-time visualization of the Li evolution and how the ASLMB behaves once a short circuit occurs (AVI)
Video S2: electro-chemo-mechanical model used to quantitatively analyze the entire process (MP4)
Notes on various aspects of our study, such as the negative to positive capacity ratios in ASLMB, Raman spectroscopic analysis of the Li-graphite layer and the solid electrolyte, *operando* neutron imaging mechanisms, normalization of neutron images, and methodologies for X-ray computed tomography analysis, modeling methodologies including Li extrusion in the Li-graphite layer, mixed ionic-electronic conduction, and simulations related to material properties, experimental methods, supplementary figures, tables, and references provide further in-depth insights (PDF)

## Supplementary Material

nl3c04072_si_001.avi

## References

[ref1] Fan L.-Z.; He H.; Nan C.-W. Tailoring inorganic-polymer composites for the mass production of solid-state batteries. Nature Reviews Materials 2021, 6 (11), 1003–1019. 10.1038/s41578-021-00320-0.

[ref2] Janek J.; Zeier W. G. A solid future for battery development. Nature Energy 2016, 1 (9), 16141 10.1038/nenergy.2016.141.

[ref3] Kamaya N.; Homma K.; Yamakawa Y.; Hirayama M.; Kanno R.; Yonemura M.; Kamiyama T.; Kato Y.; Hama S.; Kawamoto K.; et al. A lithium superionic conductor. Nat. Mater. 2011, 10 (9), 682–686. 10.1038/nmat3066. 21804556

[ref4] Adeli P.; Bazak J. D.; Park K. H.; Kochetkov I.; Huq A.; Goward G. R.; Nazar L. F. Boosting Solid-State Diffusivity and Conductivity in Lithium Superionic Argyrodites by Halide Substitution. Angew. Chem., Int. Ed. 2019, 58 (26), 8681–8686. 10.1002/anie.201814222. 31041839

[ref5] Kato Y.; Hori S.; Saito T.; Suzuki K.; Hirayama M.; Mitsui A.; Yonemura M.; Iba H.; Kanno R. High-power all-solid-state batteries using sulfide superionic conductors. Nature Energy 2016, 1 (4), 16030 10.1038/nenergy.2016.30.

[ref6] Zhang Q.; Cao D.; Ma Y.; Natan A.; Aurora P.; Zhu H. Sulfide-Based Solid-State Electrolytes: Synthesis, Stability, and Potential for All-Solid-State Batteries. Adv. Mater. 2019, 31 (44), 1901131 10.1002/adma.201901131. 31441140

[ref7] Cao D.; Sun X.; Li Q.; Natan A.; Xiang P.; Zhu H. Lithium Dendrite in All-Solid-State Batteries: Growth Mechanisms, Suppression Strategies, and Characterizations. Matter 2020, 3 (1), 57–94. 10.1016/j.matt.2020.03.015.

[ref8] Wenzel S.; Randau S.; Leichtweiß T.; Weber D. A.; Sann J.; Zeier W. G.; Janek J. Direct Observation of the Interfacial Instability of the Fast Ionic Conductor Li10GeP2S12 at the Lithium Metal Anode. Chem. Mater. 2016, 28 (7), 2400–2407. 10.1021/acs.chemmater.6b00610.

[ref9] Ji X.; Hou S.; Wang P.; He X.; Piao N.; Chen J.; Fan X.; Wang C. Solid-State Electrolyte Design for Lithium Dendrite Suppression. Adv. Mater. 2020, 32 (46), 2002741 10.1002/adma.202002741. 33035375

[ref10] Gao Y.; Wang D.; Li Y. C.; Yu Z.; Mallouk T. E.; Wang D. Salt-Based Organic-Inorganic Nanocomposites: Towards A Stable Lithium Metal/Li10GeP2S12 Solid Electrolyte Interface. Angew. Chem., Int. Ed. 2018, 57 (41), 13608–13612. 10.1002/anie.201807304. 30088847

[ref11] Zhao F.; Sun Q.; Yu C.; Zhang S.; Adair K.; Wang S.; Liu Y.; Zhao Y.; Liang J.; Wang C.; et al. Ultrastable Anode Interface Achieved by Fluorinating Electrolytes for All-Solid-State Li Metal Batteries. ACS Energy Letters 2020, 5 (4), 1035–1043. 10.1021/acsenergylett.0c00207.

[ref12] Liu G.; Weng W.; Zhang Z.; Wu L.; Yang J.; Yao X. Densified Li6PS5Cl Nanorods with High Ionic Conductivity and Improved Critical Current Density for All-Solid-State Lithium Batteries. Nano Lett. 2020, 20 (9), 6660–6665. 10.1021/acs.nanolett.0c02489. 32787073

[ref13] Lee Y.-G.; Fujiki S.; Jung C.; Suzuki N.; Yashiro N.; Omoda R.; Ko D.-S.; Shiratsuchi T.; Sugimoto T.; Ryu S.; et al. High-energy long-cycling all-solid-state lithium metal batteries enabled by silver-carbon composite anodes. Nature Energy 2020, 5 (4), 299–308. 10.1038/s41560-020-0575-z.

[ref14] Su Y.; Ye L.; Fitzhugh W.; Wang Y.; Gil-González E.; Kim I.; Li X. A more stable lithium anode by mechanical constriction for solid state batteries. Energy Environ. Sci. 2020, 13 (3), 908–916. 10.1039/C9EE04007B.

[ref15] Ye L.; Li X. A dynamic stability design strategy for lithium metal solid state batteries. Nature 2021, 593 (7858), 218–222. 10.1038/s41586-021-03486-3. 33981053

[ref16] Lee S.; Lee K.-s.; Kim S.; Yoon K.; Han S.; Lee M. H.; Ko Y.; Noh J. H.; Kim W.; Kang K. Design of a lithiophilic and electron-blocking interlayer for dendrite-free lithium-metal solid-state batteries. Science Advances 2022, 8 (30), eabq0153 10.1126/sciadv.abq0153. 35895830 PMC9328684

[ref17] Kim S. Y.; Li J. Porous Mixed Ionic Electronic Conductor Interlayers for Solid-State Batteries. Energy Mater. Adv. 2021, 1519569 10.34133/2021/1519569.

[ref18] Sole C.; Drewett N. E.; Hardwick L. J. In situ Raman study of lithium-ion intercalation into microcrystalline graphite. Faraday Discuss. 2014, 172 (0), 223–237. 10.1039/C4FD00079J. 25427224

[ref19] Gao T.; Han Y.; Fraggedakis D.; Das S.; Zhou T.; Yeh C.-N.; Xu S.; Chueh W. C.; Li J.; Bazant M. Z. Interplay of Lithium Intercalation and Plating on a Single Graphite Particle. Joule 2021, 5 (2), 393–414. 10.1016/j.joule.2020.12.020.

[ref20] Nemanich R. J.; Solin S. A.; Gúerard D. Raman scattering from intercalated donor compounds of graphite. Phys. Rev. B 1977, 16 (6), 2965–2972. 10.1103/PhysRevB.16.2965.

[ref21] Inaba M.; Yoshida H.; Ogumi Z.; Abe T.; Mizutani Y.; Asano M. In Situ Raman Study on Electrochemical Li Intercalation into Graphite. J. Electrochem. Soc. 1995, 142 (1), 20 10.1149/1.2043869.

[ref22] Nikiel L.; Jagodzinski P. W. Raman spectroscopic characterization of graphites: A re-evaluation of spectra/ structure correlation. Carbon 1993, 31 (8), 1313–1317. 10.1016/0008-6223(93)90091-N.

[ref23] Irish D. E.; Deng Z.; Odziemkowski M. Raman spectroscopic and electrochemical studies of lithium battery components. J. Power Sources 1995, 54 (1), 28–33. 10.1016/0378-7753(94)02035-2.

[ref24] Wang H.; Ning D.; Wang L.; Li H.; Li Q.; Ge M.; Zou J.; Chen S.; Shao H.; Lai Y.; et al. In Operando Neutron Scattering Multiple-Scale Studies of Lithium-Ion Batteries. Small 2022, 18 (19), 2107491 10.1002/smll.202107491. 35195340

[ref25] Doux J.-M.; Nguyen H.; Tan D. H. S.; Banerjee A.; Wang X.; Wu E. A.; Jo C.; Yang H.; Meng Y. S. Stack Pressure Considerations for Room-Temperature All-Solid-State Lithium Metal Batteries. Adv. Energy Mater. 2020, 10 (1), 1903253 10.1002/aenm.201903253.

[ref26] Liu W.; Luo Y.; Hu Y.; Chen Z.; Wang Q.; Chen Y.; Iqbal N.; Mitlin D. Interrelation Between External Pressure, SEI Structure, and Electrodeposit Morphology in an Anode-Free Lithium Metal Battery. Adv. Energy Mater. 2023, 2302261 10.1002/aenm.202302261.

[ref27] Wang Y.; Liu Y.; Nguyen M.; Cho J.; Katyal N.; Vishnugopi B. S.; Hao H.; Fang R.; Wu N.; Liu P.; et al. Stable Anode-Free All-Solid-State Lithium Battery through Tuned Metal Wetting on the Copper Current Collector. Adv. Mater. 2023, 35 (8), 2206762 10.1002/adma.202206762. 36445936

[ref28] Liu Z.; Han G.; Sohn S.; Liu N.; Schroers J. Nanomolding of Crystalline Metals: The Smaller the Easier. Phys. Rev. Lett. 2019, 122 (3), 036101 10.1103/PhysRevLett.122.036101. 30735412

[ref29] Barroso-Luque L.; Tu Q.; Ceder G. An Analysis of Solid-State Electrodeposition-Induced Metal Plastic Flow and Predictions of Stress States in Solid Ionic Conductor Defects. J. Electrochem. Soc. 2020, 167 (2), 020534 10.1149/1945-7111/ab6c5b.

[ref30] Cabañero M. A.; Boaretto N.; Röder M.; Müller J.; Kallo J.; Latz A. Direct Determination of Diffusion Coefficients in Commercial Li-Ion Batteries. J. Electrochem. Soc. 2018, 165 (5), A847 10.1149/2.0301805jes.

[ref31] Biswal P.; Stalin S.; Kludze A.; Choudhury S.; Archer L. A. Nucleation and Early Stage Growth of Li Electrodeposits. Nano Lett. 2019, 19 (11), 8191–8200. 10.1021/acs.nanolett.9b03548. 31566985

[ref32] Pei A.; Zheng G.; Shi F.; Li Y.; Cui Y. Nanoscale Nucleation and Growth of Electrodeposited Lithium Metal. Nano Lett. 2017, 17 (2), 1132–1139. 10.1021/acs.nanolett.6b04755. 28072543

