# Supplementary material for: Li Dynamics in Mixed Ionic-Electronic Conducting Interlayer of All-Solid-State Li-metal Batteries
Source: Nano Lett. 2024 Jan 25;24(5):1544–52. doi: 10.1021/acs.nanolett.3c04072 (PMC10853963; doi:10.1021/acs.nanolett.3c04072)
Supplement: Supplementary file 3 — nl3c04072_si_003.pdf [file nl3c04072_si_003.pdf]

Supplementary Information for

Li Dynamics in Mixed Ionic-electronic Conducting Interlayer of All-solid-state Li-metal Batteries

Daxian Cao[1], Yuxuan Zhang[2], Tongtai Ji[1], Xianhui Zhao[3], Ercan Cakmak[4], Soydan Ozcan[5], Michael Geiwitz[6], Jean Bilheux[2], Kang Xu[7], Ying Wang[1], Kenneth Stephen Burch[6,], Qingsong Howard Tu[8,*], Hongli Zhu[1,*]*

[1]Department of Mechanical and Industrial Engineering, Northeastern University, Boston, Massachusetts 02115, USA

[2]Neutron Scattering Division, Oak Ridge National Laboratory, Oak Ridge, Tennessee 37831, USA

[3]Environmental Sciences Division, Oak Ridge National Laboratory, 1 Bethel Valley Road, Oak Ridge, Tennessee 37830, United States

[4]Materials Science and Technology Division, Oak Ridge National Laboratory, Oak Ridge, Tennessee 37831, USA

[5]Manufacturing Science Division, Oak Ridge National Laboratory, 1 Bethel Valley Road, Oak Ridge, Tennessee 37830, United States

[6]Department of Physics, Boston College, Chestnut Hill, Massachusetts 02467, USA

[7]Battery Science Branch, Sensor and Electron Devices Directorate, CCDC Army Research Laboratory, Adelphi, Maryland 20783-1197, USA

[8]Mechanical Engineering, Rochester Institute of Technology, Rochester, NY 14623, USA

***Corresponding author.** h.zhu@neu.edu; howard.tu@rit.edu; burchke@bc.edu

Contents

Note S1. The negative to positive capacity ratios in ASLMB. 3
Note S2. Raman spectra revealing the composition of the Li-graphite layer 3
Note S3. Raman spectra of the SE 3
Note S4. Operando Neutron imaging mechanism 3
Note S5. Normalization of the raw neutron images 3
Note S7 Quantify the neutron transmission at different positions 4
Note S8 Normalization of the neutron transmission to the initial state 4
Note S9. Second operando neutron imaging test 5
Note S10. Operando Raman spectroscopy 6
Note S11. X-ray computed tomography (XCT) analysis 6
Note S12. Modelling methodologies 7
1) Modeling of Li extrusion inside the Li-graphite layer 7
2) Mixed ionic-electronic conduction of the Li-graphite layer 8
3) Simulations and material properties 9
Note S13: Experimental methods 10
Supplemental figures (Fig. S1 to S23) 15
Supplemental Tables 29
References 29

Note S1. **The negative to positive capacity ratios in ASLMB.**

Since graphite is the most used anode material in lithium ion batteries, the negative to positive capacity ratio is critical to determine the role of graphite as anode material or interlayer. In our work, the capacity ratio of the negative to positive electrodes was 2.26 based on the total theoretical capacity of graphite plus Li and 0.43 based solely on graphite to guarantee the graphite plays the role of interlayer.

Note S2. Raman spectra revealing the composition of the Li-graphite layer

The pristine graphite shows a sharp peak at 1580 cm^{-1} (G band) assigned to the in-plane C-C stretching and a weak peak at 1340 cm^{-1} (D band), indicating minor disorder.[1] In Li-graphite, the G peak shifts to 1593 cm^{-1}, and a broad feature near 1350 cm^{-1} appear. The blue shift of the G peak suggests the intercalation of Li into graphite forming GIC.[2] In our work, we used the G peak and the broad feature evolution to track the Li behaviors of intercalation and plating at the interlayer.

Note S3. Raman spectra of the SE

The peaks at 427, 577, and 600 cm^{-1} are attributed to the PS_4^{3-} unit in the SE, $Li_{5.4}PS_{4.4}Cl_{1.6}$, which owns a typical argyrodite structure.

Note S4. Operando Neutron imaging mechanism

The neutron beam goes through the specially designed cell (**Fig. S20**), and the scintillators convert the neutrons into light signals. The detector then records the signal to construct the gray-scale radiography. Such neutron radiographs are collected continuously while a cell is running. The Li concentration evolution can be real-time observed by tracking the attenuation change.

Note S5. Normalization of the raw neutron images

The raw image experienced a normalization treatment to enhance the signal-to-noise ratio. The first step is to remove saturated pixels and backgrounds. Then every 30 continuous images are

combined to enhance the signals. Since the image is taken every 10 s, the time resolution of the image is 5 min after treatment. All these treatments are conducted with the Jupyter Imaging Notebook developed by ORNL (https://neutronimaging.ornl.gov/tutorials/imaging-notebooks/).

Note S6. 2D Neutron radiography of the ASLMB before cycling

Fig. 4a shows the neutron radiography image of the ASLMB before cycling. Based on the neutron attenuation differences, each layer can be clearly identified, including Li-metal anode, graphite layer, SE, and cathode from the top to bottom. Notably, metallic Li and Li-containing materials show darker colors owing to their high neutron attenuation coefficient.

Note S7 Quantify the neutron transmission at different positions

Theoretically, the total neutron transmission (T) was defined as the ratio of transmitted beam intensity (I) and incident beam intensity (I_0), which is determined by the attenuation coefficient (μ) and sample thickness (x) as the following equation,

$$T = \frac{I}{I_0} = e^{-\mu x}$$

The neutron transmission, $I_N(i, j)$, can be normalized in the range from 0 to 1 through

$$I_N(i,j) = \frac{I(i,j) - DF(i,j)}{OB(i,j) - DF(i,j)}$$

where $I(i, j)$, $OB\ (i, j)$, and $DF\ (i, j)$ represent the raw neutron intensity, open beam intensity, and dark field intensity, respectively.

Note S8 Normalization of the neutron transmission to the initial state

The 2D neutron radiography reveals the sum of neutron transmission information in one view. Therefore, the evolution of the Li is imperceptible due to the low contrast. However, we can normalize the neutron transmission (Tr_t) at the battery charging time (t) to the initial transmission (Tr_0) through a division treatment to get the transmission change ratio ($\mathrm{Tr}_t/\mathrm{Tr}_0$). Then the $\mathrm{Tr}_t/\mathrm{Tr}_0$

can be used to evaluate the Li evolution. The enhanced neutron transmission ($Tr_t/Tr_0>1$) shows a bright spot on the image representing the reduction of Li; the reduced transmission ($Tr_t/Tr_0<1$) exhibits a dark spot meaning enriched Li; the stable transmission ($Tr_t/Tr_0=1$) is depicted as gray color suggesting no Li change. This treatment is conducted with the Jupyter Imaging Notebook developed by ORNL.

Note S9. Second operando neutron imaging test

To validate that our original observation was not an anomalous result, an additional operando neutron imaging experiment was conducted. As shown in **Fig. S14**, this cell completed a full charge-discharge cycle without failure. The cell exhibited high initial resistance (**Figs. S14a,b**), reaching the cut-off voltage around 4.5 hours at a C/20 rate. To ensure sufficient Li migration, the charge protocol was modified from constant current to constant voltage charging up to 20 hours (**Fig. S14c**). This yielded a total charge capacity of 76 mAh g=1 (1.2 mAh cm-2 of Li). The cell was then discharged at C/20 to 2.8 V and constant voltage at 2.8 V to total 20 hours, delivering 55 mAh g-1 (0.87 mAh cm-2 of Li).

The cross-sectional Li concentration profile was obtained from the neutron transmission (**Fig. S14d**) and used to identify the interfaces between components (**Fig. S14e**). Voltage-time curves over the full cycle are shown in **Fig. S14f**. The Li distribution evolution indicates a stepped deposition process on charge, with Li first accumulating at the Li-Gr|SE interface and in the Li-Gr interlayer (**Fig. S14g**). On discharge, stripping occurred primarily from the interface region and Li-Gr. This additional result is consistent with our previous operando data, providing further evidence that Li preferentially deposits atop rather than below the graphite interlayer. The reproducibility supports our conclusions.

Note S10. Operando Raman spectroscopy

A key challenge in operando Raman spectroscopy is the air sensitivity of the ASLMB. It typically requires a quartz window to seal the cell, which reduces the Raman intensities. Thus we designed an *in-situ* Raman cell (**Fig. 1c**) and operated the test inside an Argon-filled glovebox[3] (**Fig. S22)**. The inert atmosphere allows the ASLMB to be measured without a sealing setup. There is a window on the cell side that can be removed after cell assembly (**Fig. S23**) to make sure a high pressure stacking in cell assembly and a total exposure of the cross-section surface to the laser during the test. An outside framework enables controllable pressure on the cell during the test, to guarantee the operando test is at the right electrochemical reaction states. Also, the square-type cell obtains a flat surface avoiding the noise caused by sample flatness, which will affect both the electrochemical reaction and optical light focus. A line scan along the cross section can well detect the evolution of Li-graphite at different positions.

Note S11. X-ray computed tomography (XCT) analysis

The ASLMB for the XCT test was assembled in a homemade cell (**Fig. S18**) which was used for the electrochemical test first and then directly applied in XCT measurement without sample distracting process. **Fig. S18a** schematically illustrates the cell configuration and the XCT test process. Cathode, SE, graphite, and Li metal are layer-by-layer stacked together in the cell, and two stainless steel rods work as the current collector. The X-ray beams transmit through the sample and are converted into optical signals which are then collected for grayscale radiograph construction. The sample stepwise rotates and several Slices can be obtained. Then the 3D structure of the sample can be reconstructed. After 1.1 hours of plating at the rate of C/20, the cell shows a typical soft short circuit and maintains until 20 hours (**Fig. S18b**). After reconstruction, we can obtain the 3D structure of the ASLMB after the short circuit**.**

The laminated structure of the ASLMB is well revealed in the reconstructed structure, as shown in **Fig. S18c**. The grayscale contrast is caused by the density difference. As a result, the cathode active material owns the most X-ray absorption, showing the highest brightness, In comparison, sulfide SE shows lower brightness, while Li metal, as well as the voids, behaves as the deepest darkness. Five representative slices are extracted to show the details inside the SE.

Figures S18d and e display Slices 1 and 2 in the cross-section of the ASLMB, respectively. We can see the densely packed cathode, SE, and Li metal. There are some tiny black spots inside the SE that can be assigned to the voids formed in the cold pressing process. More significantly, there are filaments with dark color grown from the anode towards the cathode. **Figures S18f-h** show Slices 3, 4, and 5 from the top view. There are also a few dark filaments observed.

These filaments explain the failure of the ASLMBs. According to the highest X-ray attenuation, these filaments can be attributed to Li metal within cracks. During plating, the Li metal deposits onto the Li-graphite and has direct contact with the SE. [4, 5]. The Li metal within the cracks propagates and finally penetrates the SE resulting in the short circuit. This is one of the most reported failure mechanisms of the ASLMB. Nevertheless, the origin of this phenomenon is the Li metal deposits above the Li-graphite interlayer. The battery performance and the Li behaviors will be different if the Li metal deposits beneath the Li-graphite.

Note S12. Modelling methodologies

1) Modeling of Li extrusion inside the Li-graphite layer

The extrusion of Li within the pores of the Li-Gr layer has been reported in many other work[6-8]. At such high pressure ($P_0 = 300MPa$), Li metal is under severe plasticity and can be simulated effectively using computational fluid dynamics, where the material is considered as a fluid with a very high viscosity that depends on velocity and temperature.

The key parameter pseudo-viscosity η is obtained following the Perzyna formulation of strain rate for associated flow[9], using Von-Mises type flow. The pseudo-viscosity can be expressed in terms of the yield stress σ_Y of the deforming metal, a plasticity relaxation rate γ, and the effective plastic strain rate $\dot{\bar{\epsilon}}$, which is compactly expressed using the inner product, $\dot{\bar{\epsilon}} = \sqrt{2\dot{\epsilon_p}:\dot{\epsilon_p}}$,

$$\eta = \frac{\frac{\sigma_Y}{\sqrt{3}} + \left(\frac{\dot{\bar{\epsilon}}}{\gamma}\right)^n}{\dot{\bar{\epsilon}}}$$

The hardening exponent n quantifies the degree of work-hardening during deformation. All the involved parameters of Li metal can be found in earlier work[10].

2) Mixed ionic-electronic conduction of the Li-graphite layer

If the graphite layer is treated as a MIEC, the transport of Li^+ and electrons inside it can be described by the ohmic relations:

$$\boldsymbol{i}_{Li^+} = -\frac{\sigma_{Li^+}}{F}\nabla\tilde{\mu}_{Li^+}, \qquad \boldsymbol{i}_{e^-} = \frac{\sigma_{e^-}}{F}\nabla\tilde{\mu}_{e^-}. \tag{1}$$

The ionic and electronic current density in the SE ($\boldsymbol{i}_{Li^+}$, $\boldsymbol{i}_{e^-}$), the electrochemical potential of Li^+ in the Li-graphite ($\tilde{\mu}_{Li^+}$), and the electrochemical potential of e^- in the Li-graphite ($\tilde{\mu}_{e^-}$). The electrochemical potentials defined here are the Gibbs energies (in energy per mole of Li^+ cations) for a monovalent reaction ($Li^+ + e^- \leftrightarrow Li$), including the effects on electric potentials. $\tilde{\mu}_{Li^+}$ is related to the electrochemical potential of electrons in electrodes ($\tilde{\mu}_{e^-}$) and the chemical potential of Li in the Li-graphite layer (μ_{Li}) through the relation $\tilde{\mu}_{Li^+} + \tilde{\mu}_{e^-} = \mu_{Li}$.

It is assumed here that the partial current densities of cations ($\boldsymbol{i}_{Li^+}$) and electrons ($\boldsymbol{i}_{e^-}$) are only dependent on their respective gradients in electrochemical potentials, $\tilde{\mu}_{Li^+}$ and $\tilde{\mu}_{e^-}$, respectively. The cross-effect of one electrochemical potential on the other's partial current has been omitted in this study because of their second-order contributions. The partial conductivities σ_{Li^+} and σ_{e^-} are defined under Ohmic current flow and are assumed to be constants in this study.

Both the electrochemical potential of Li^{+} ($\tilde{\mu}_{Li^{+}}$) and electrons ($\tilde{\mu}_{e^{-}}$) should follow purely Ohmic conduction, as described by the Nernst-Planck equation:

$$\frac{\partial c_{Li^{+}}}{\partial t} = -D_{Li^{+}} \nabla^{2} C_{Li^{+}} - \sigma_{Li^{+}} \nabla^{2} \tilde{\mu}_{Li^{+}}.$$

3) Simulations and material properties

Multiple physics are involved and coupled from the mechano-chemical to the electro-chemical process, which are described by highly nonlinear partial differential equations (PDEs). A self-developed code based on the finite element method and the MOOSE framework[11] was implemented to solve all PDEs numerically.

The default values of the parameters used in this work from reported experimental measurements in reference papers are listed in the last column in **Table S2**.

Note S13: Experimental methods

Preparation of materials

The sulfide solid electrolyte, $Li_{5.4}PS_{4.6}Cl_{1.4}$, was synthesized through a conventional ball milling and annealing process. Lithium sulfide (Li_2S, 99.98%, Sigma-Aldrich), phosphorus pentasulfide (P_2S_5, 99%, Sigma-Aldrich), and lithium chloride (LiCl, >99.98%, Sigma-Aldrich) were stoichiometrically mixed in a stainless steel jar under vacuum for 10 h at 500 rpm. ZrO_2 balls were added in a weight ratio of 50:1. The mixture was then sealed in a glass tube and heat treated at 510 °C for 2 h.

The cathode active material used in this work was a single crystal $LiNi_{0.8}Mn_{0.1}Co_{0.1}O_2$ (NMC, Nanoramic Inc., USA). To avoid an interface reaction with the sulfide solid electrolyte, the NMC particle was coated with a layer of amorphous Li_2SiO_x using a reported wet-chemical method. Then, the cathode was prepared by mixing the Li_2SiO_x-coated NMC, $Li_{5.6}PS_{4.6}Cl_{1.4}$, and VGCF in a ratio of 80:20:2 in a ball-milling jar under vacuum for 30 min at 150 rpm.

The graphite (Gr, 99% purity, Alfa Aesar) was dried overnight at 100°C under vacuum prior to use. To prepare the graphite interlayer film, 98 mg of the dried graphite powder was mixed with 2 mg of freeze-dried polytetrafluoroethylene (PTFE, 60 wt% in H2O, Sigma-Aldrich) binder. The mixing was performed at 200°C to disperse the PTFE throughout the graphite. This composite mixture was pressed into flakes and then rolled into a thin, free-standing film with a thickness of 14-30 μm. The Li metal (99.9%, Sigma-Aldrich) was first scratched with a blade to remove the surface oxidation layer and then pressed into a thin film with a thickness of 100 μm.

Symmetric cell fabrication

The symmetric cell was fabricated in a conventional pressure cell. 150 mg SE was densified inside a polyether ether ketone (PEEK)-made cell with two stainless-steel rods of a diameter of 12.7 mm. On each side, one piece of graphite layers (~4 mg, 12.6 mm in diameter, 25 μm) and one piece of

Li layer (~2 mg, 10 mm in diameter) are pressed onto the SE pellet in sequence. High pressure of 300 MPa was applied to the cell for 3 min by a hydraulic press. Then the pressure was released and an initial pressure of 100 MPa was applied to the cell during the cell operation by a stainless steel framework. The cell was measured at varied current densities of 0.25, 1, 2, 4, and 10 mA cm^{-2} at a fixed charge of 0.25 mAh cm^{-2}.

Full cell fabrication

The full cells were assembled in a standardized pressure cell setup allowing direct comparison between different graphite interlayer thicknesses. First, 150mg of solid electrolyte (SE) powder was densified at 300 MPa for 3min. Then a graphite interlayer of varying thickness was placed on the SE pellet followed by a 10mm diameter Li foil (~2mg). This Li-graphite stack was pressed onto one side of the SE pellet at 300 MPa. On the other side, 25mg of cathode powder was cast directly onto the SE pellet surface. The cell was sealed and an equilibrium pressure of 10 MPa was maintained by the rigid framework. This process kept all variables constant besides the thickness of the graphite interlayer film.

The preparation of LiC_6 through the electrochemical method

The LiC6 was prepared electrochemically in a CR2032 coin cell with a liquid electrolyte. A 10 mm diameter freestanding graphite film served as the working electrode, while Li metal was used as the counter and reference electrode. 80 μL of 1.2 M $LiPF_6$ in 3:7 weight ratio EC:EMC (Gen 2 electrolyte) was added along with a Celgard 2325 separator. The cell was assembled entirely inside an Ar-filled glovebox. To lithiate the graphite, the cell was discharged at a C/50 rate from open circuit voltage down to 0.01 V. The resulting LiC_6 electrode was then extracted from the cell and rinsed with dimethyl carbonate to remove residual electrolyte salt and solvent. After drying

overnight under vacuum in the glovebox to fully evaporate the solvent, the LiC_6 electrode was analyzed by a Raman spectroscopy in the glovebox.

Design of the cell for operando neutron imaging

The design of the cell for *operando* neutron imaging, as illustrated in Fig. 1a in the main text, was based on the pressure cell used in most studies on sulfide-SE-based ASLMBs. Two stainless-steel rods with 6.35-mm diameter were used as the current collectors and pressing plugs. The ASLMB was directly fabricated in a PTFE tube with a 9.53-mm outside diameter and 6.35-mm inside diameter. In further detail, 60 mg of $Li_{5.6}PS_{4.6}Cl_{1.4}$ was pressed into a pellet inside the PTFE tube under a pressure of 300 MPa by a hydraulic press. Then, 8 mg of the cathode powder was cast and pressed onto one side of the pellet at 100 MPa. After that, a piece of graphite layer (~1.5 mg, 5.56 mm in diameter) and a piece of Li layer (~0.6 mg, 3.97 mm in diameter) were pressed onto the other side in sequence. The cell was then pressed at 300 MPa for 3 min. After releasing the pressure, the cell was fixed in a stainless steel framework by tighten the screw nuts with a calibrated torque, which applied an initial pressure of 10 MPa to the cell.

Design of the cell for operando Raman

The design of the *operando* Raman cell is illustrated in Fig. 1b in the main text. The ASLMB was assembled in a PEEK-made holder. Unlike the cylindrical morphology of the neutron imaging cell, the Raman cell was designed with a cubic morphology, providing a cross section with a flat surface. One side of the PEEK could be opened after the cell assembly to directly expose the samples to the laser. For the cell assembly, 60 mg of $Li_{5.6}PS_{4.6}Cl_{1.4}$ was pressed into a 6×6 mm^2 pellet first in the PEEK holder at 300 MPa by a hydraulic press. Then, 8 mg of the cathode powder was cast and pressed onto one side of the pellet at 100 MPa by a hydraulic press. Next, a piece of graphite (~1.5 mg, 5×5 mm^2) and a piece of Li (~0.6 mg, 3×4 mm^2) were pressed onto the other

side. The total cell was pressed together at 300 MPa for 3 min. After releasing the pressure, the cell within the framework was applied an initial pressure of 10 MPa enabled by tightening screws with a torque wrench.

Operando neutron imaging experiment

The *operando* neutron imaging experiment was performed at the cold neutron imaging beamline, CG-1D, at the High Flux Isotope Reactor, Oak Ridge National Laboratory. The neutron beam is a polychromatic cold neutron beam with a wavelength range from 0.8 to 6 Å and a peak intensity of 2.2×10^{6} n cm^{-2} s^{-1} at 2.6 Å. The detector-to-pinhole distance, *L*, is 6.59 m, and the pinhole size, *D*, is 11 mm, resulting in a spatial resolution of ~25 µm. The neutron attenuation in the ASLMB was collected by a scientific complementary metal-oxide-semiconductor (sCMOS) camera system (Zyla5.5, Andor Technology plc. Belfast, UL) with a 20-µm-thick Gd_2O_2S:Tb scintillator screen. During the battery charging, the neutron radiographs were collected every 10 s to achieve the time-stamped continuous transmission change. Before and after the battery test, the background radiographs, including the dark-field measurements with beam off and open beam measurements with a flat field, were collected for further image normalization.

Operando Raman experiment

The *operando* Raman experiment was performed at Boston College. In detail, the as-assembled ASLMB was sealed well in the glovebox (oxygen and water contents below 0.01 and 0.01 ppm, respectively) and then transferred into the glovebox (oxygen and water contents below 1 and 0.1 ppm, respectively) where the Raman was installed. The Raman test was performed with WITec. The laser wavelength was 532 nm, and the laser power was 200 mW. The cell was horizontally installed in the Raman instrument, and the sample was directly exposed to the laser. The cables connected to both positive and negative electrodes of the cell were extended out of the glovebox

to connect with the electrochemical workstation (Biologic SP150). During the test, the cell automatically moved along the line across the anode interface. The Raman was taken every 5 µm and 6 points in total. The beam size was around 1 µm. The total time for the collection of one spectrum was 25 s. Therefore, the total time for one scan was 150 s. The spectra were collected every 10 min.

Ex-situ XCT test

The ASLMB used for XCT test was assembled in a home-made cell with a diameter of 2 mm inner diameter. 15 mg of SE was densified inside the tube first by two stainless steel rods under axial pressure. 1 mg of cathode powder was cast onto one side. A piece of graphite was pressed onto Li foil first and then punched into a 2 mm disc. The Li-graphite disc was attached to the other side. Two stainless steel rods were fixed onto the PEEK tube through a screw connection, which could apply external stacking pressure to the battery during the test. The battery was measured at C/20. After the battery test, the cell was directly used for the XCT test without other treatment.

The XCT data collection was conducted using a Zeiss Xradia Versa 520 XCT unit, operated at 60 kV and 83.6 µA. The XCT data were acquired from a sample rotation of 360° (vertical axis) with 1601 projections at equal steps. A 1 × 1 binning on the detector side was used to maximize resolution. The voxel size was 1.62 µm with the 4x objective. For image processing and segmentation, the Fiji software v. 2.9.0 and ORS Dragonfly PRO v.3.5 software were used.”.

Supplemental figures (Fig. S1 to S23)

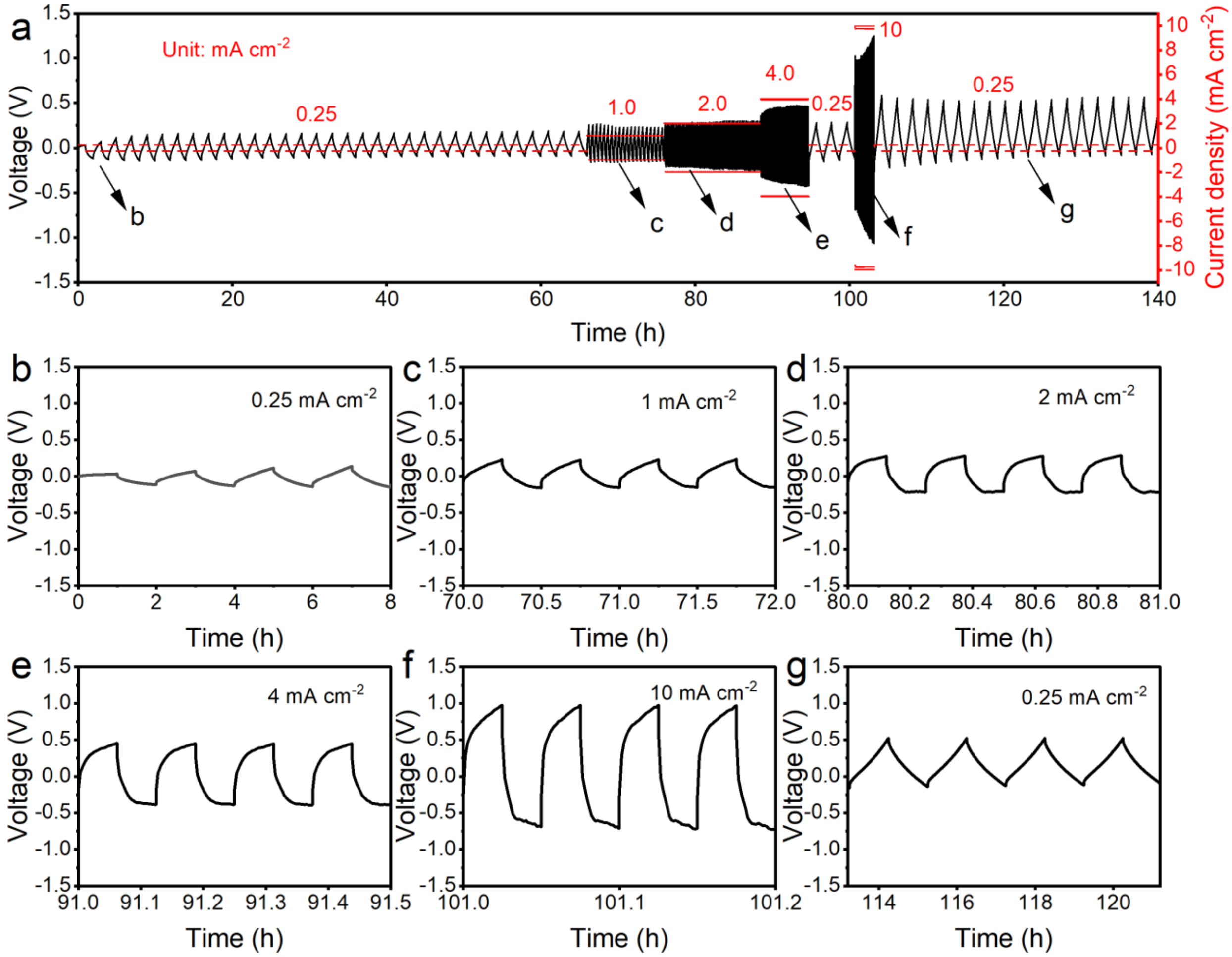

Fig. S1. **a.** Cycling stability of Li-Gr stabilized Li metal anode in the Li^0/Li^0 symmetric cell. **b-d** Detailed profiles of the cell cycled at current densities of 0.25 **b)** , 1 **c)**, 2 **d)**, 4 **e)**, 10 **f)**, and a second round of 0.25 **g)** mA cm^{-2}.

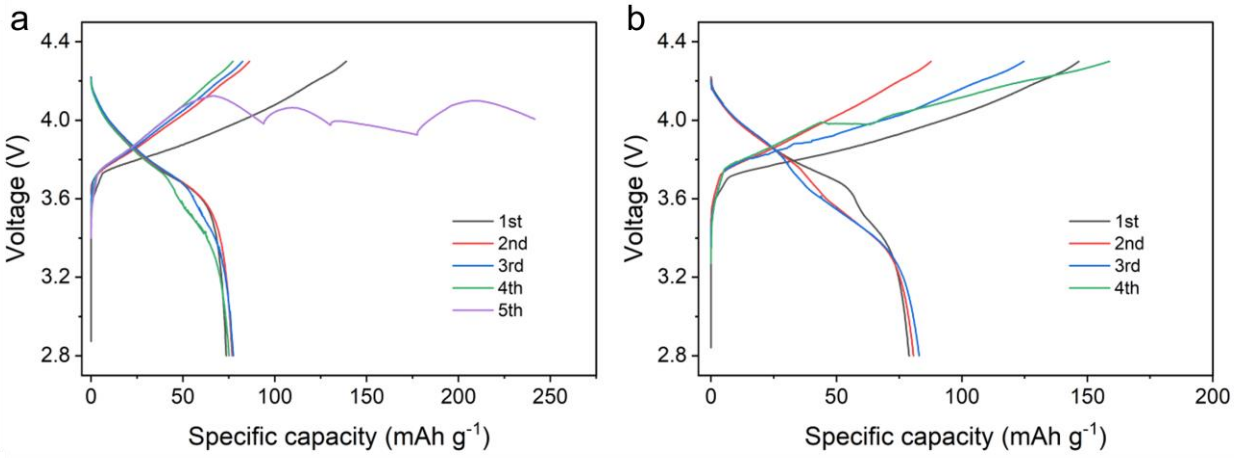

Fig. S2. Galvanostatic charge profile of the full cell using graphite interlayer with thicknesses of 17 μm **a)** and 14 μm **b)**.

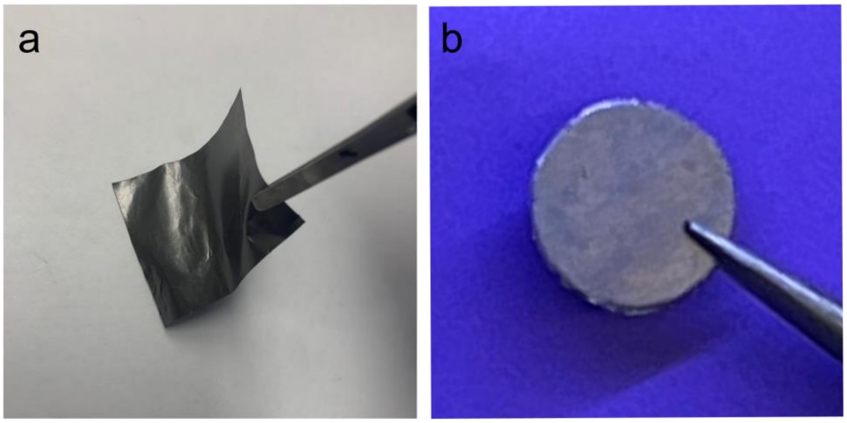

Fig. S3. Photos of (a) the freestanding graphite layer and (b) Li-graphite on the top view. The black color evidence the Li-graphite was not fully intercalated.

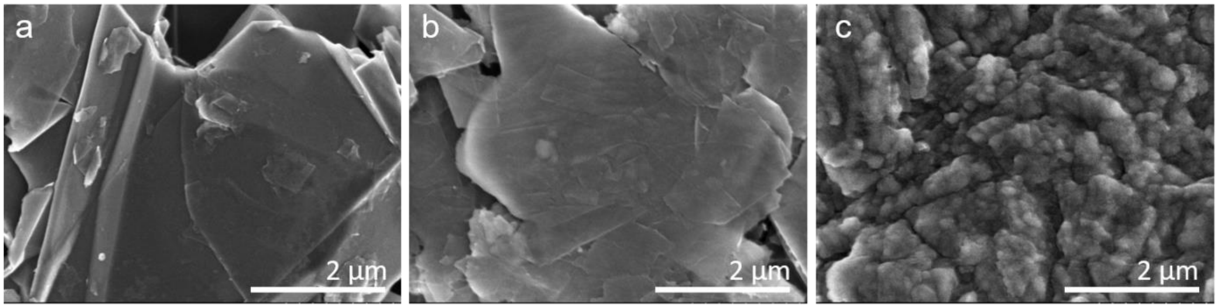

Fig. S4. Morphology investigation. a, SEM images of the Gr powder **b,** SEM images of the Gr layer. **c,** SEM images of the Li-Gr.

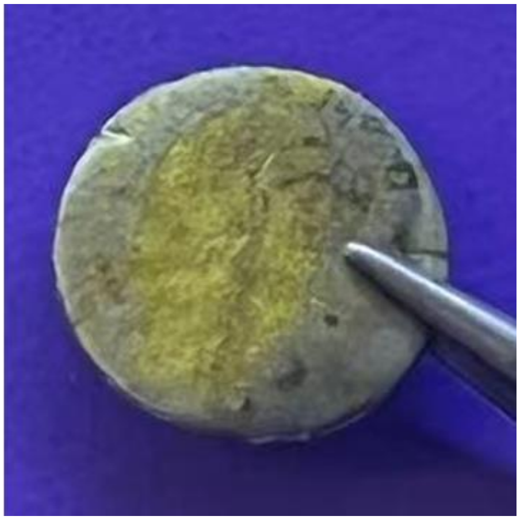

Fig. S5. Photo of the Li-Gr after heating at 160 C. The gold color demonstrates the formation of LiC_6.

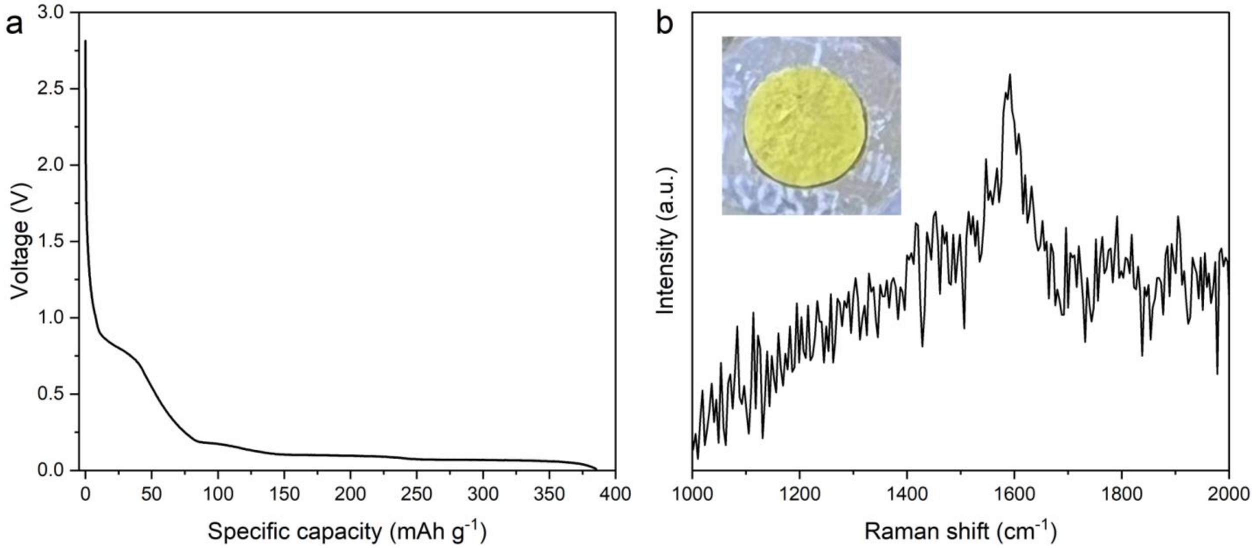

Fig. S6 a. The lithiation of graphite in the cell using liquid electrolyte. **b.** The Raman spectrum of the LiCx prepared through lithiation in liquid electrolyte. The inset photo shows the fully lithiated graphite, which has a golden color.

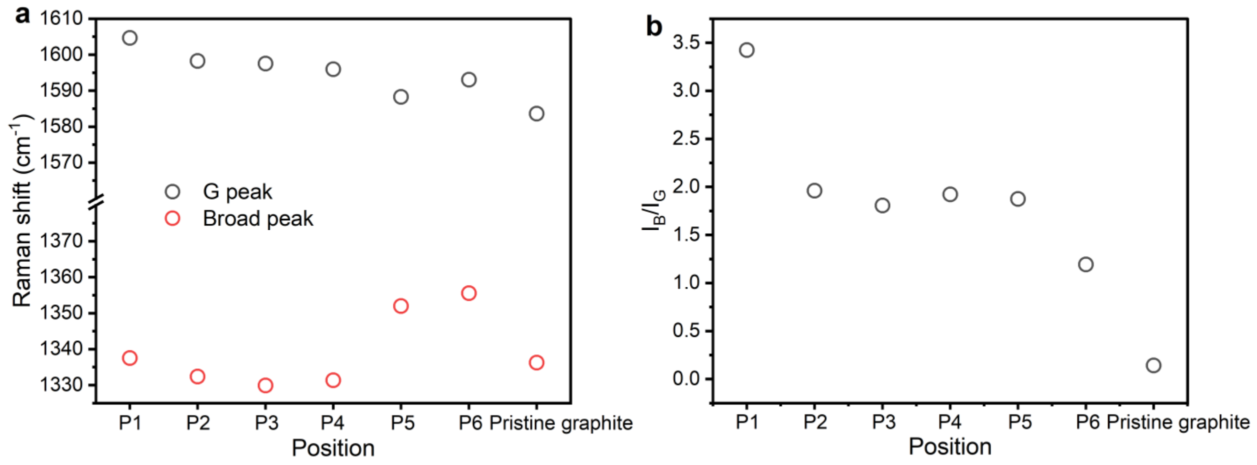

Fig. S7. a. Raman shifts of broad feature and G peak at different locations in Li-graphite. **b**. I_B/I_G ratio comparison among different locations. The pristine graphite was also list for reference.

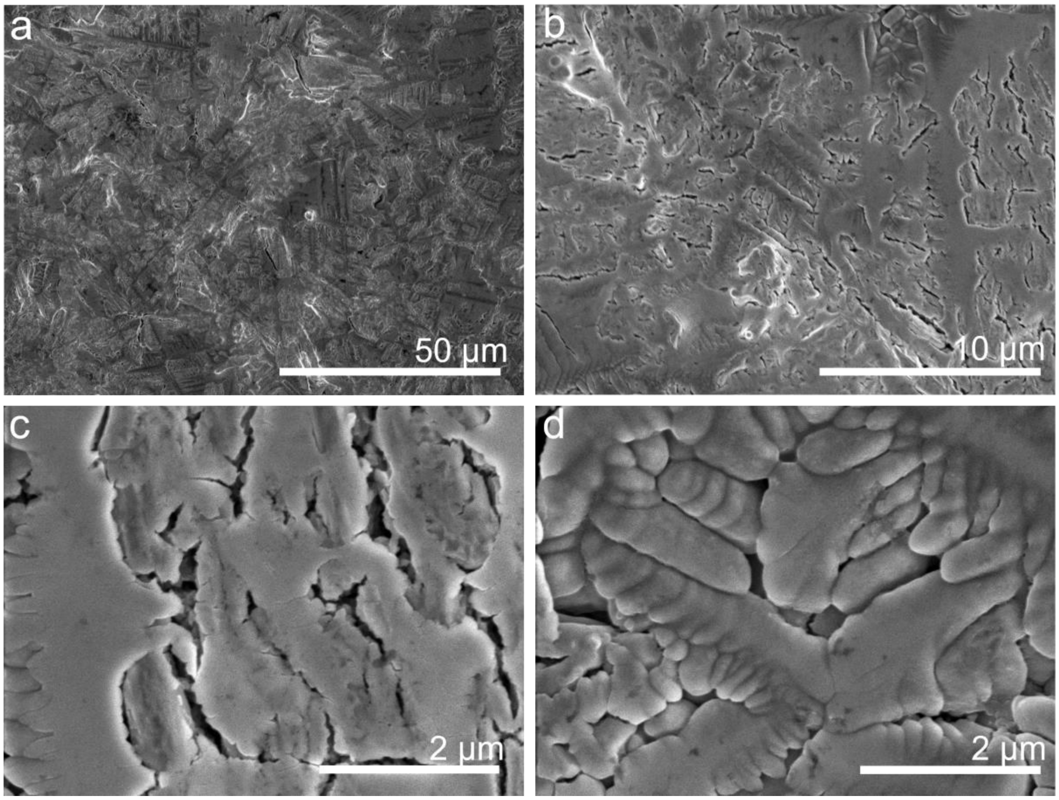

Fig. S8. Morphology of the SE surface after cold pressing in magnification of (a) 1k x, (b) 5k x (c) 20k x, and (d) 20k x.

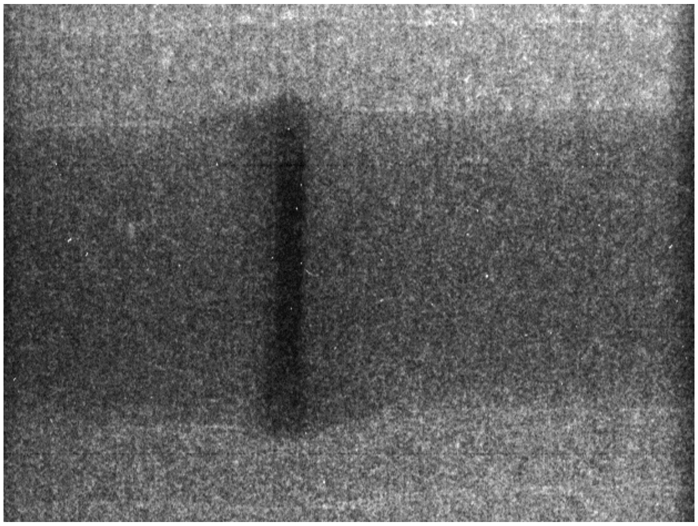

Fig. S9. Raw radiograph image of the cell before test.

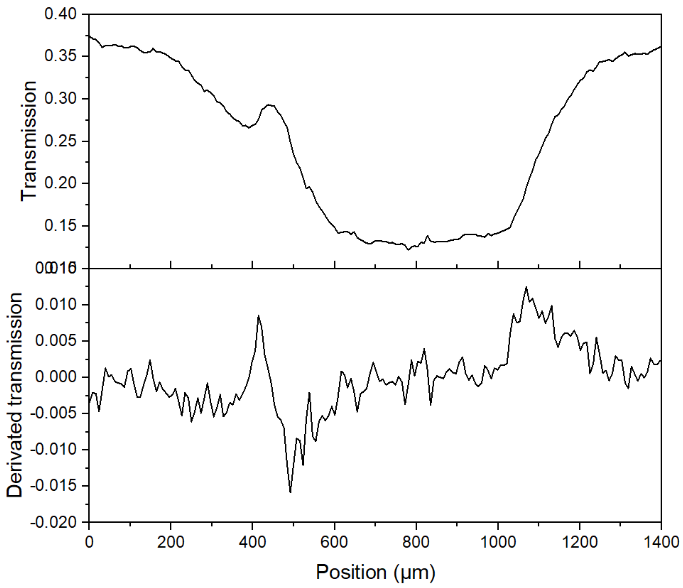

Fig. S10. Neutron transmission and derivated transmission to determine the interfaces between adjacent layers.

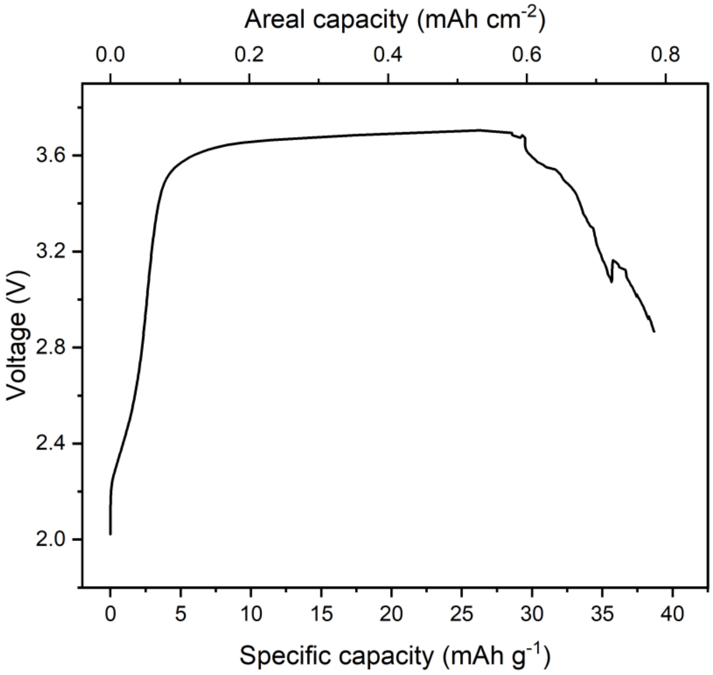

Fig. S11. Galvanostatic charge profile of the full cell in the operando neutron test.

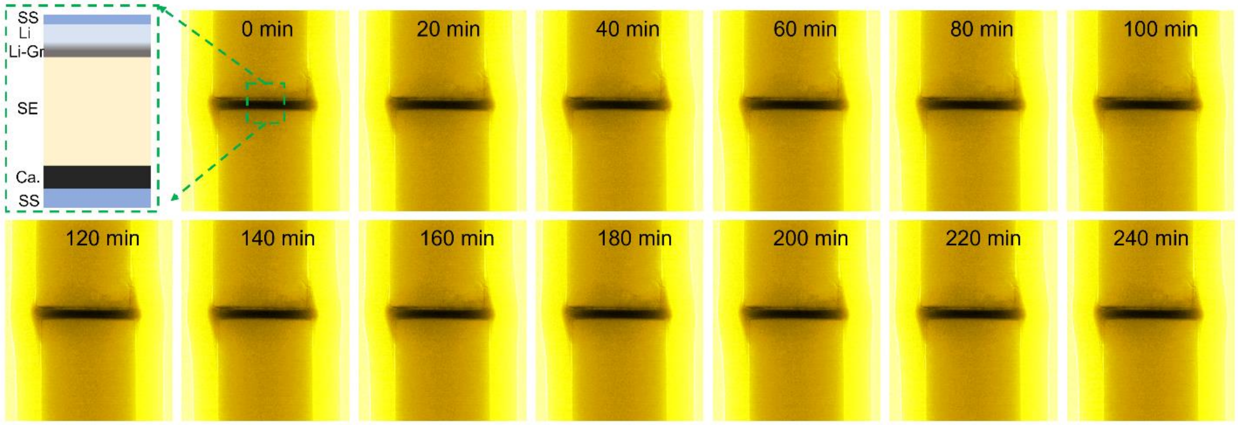

Fig. S12. Time stamped neutron radiography images of the ASLMB during test. The transmission change is imperceptible. The inset schematic illustrates the architecture of the ASLMB.

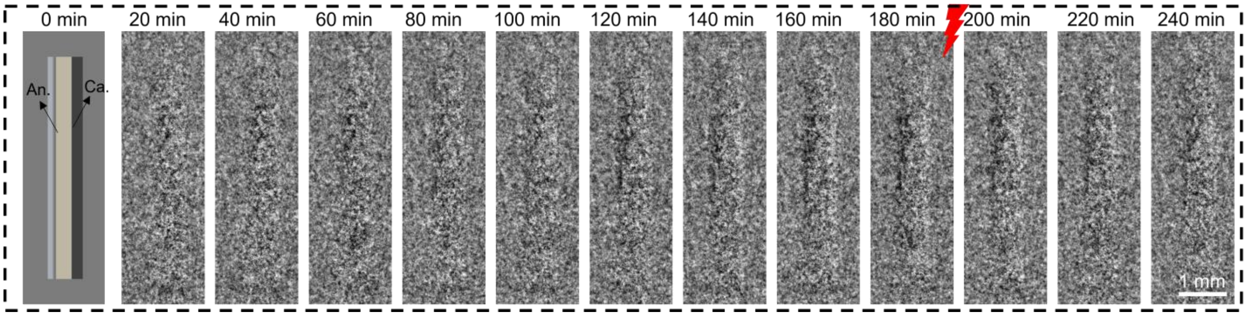

Fig. S13. Neutron radiography images normalized to the pristine state show the transmission change ratio (Tr_t/Tr_0) as a function of charging time. The dark and bright regions in the figures depict the enriched Li and Li depletion, respectively. The red lightning symbol indicates a short circuit.

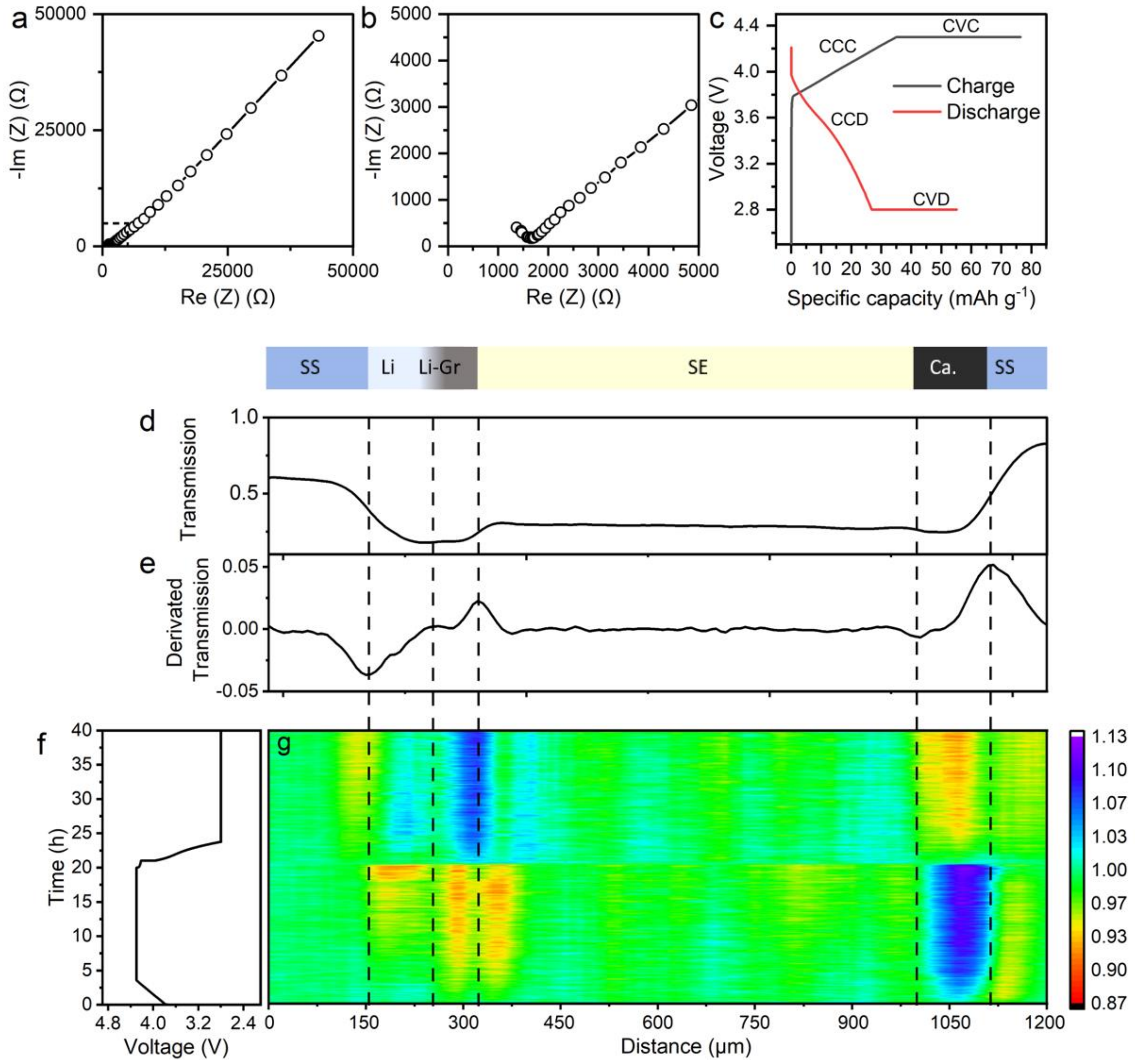

Fig. S14. Operando neutron imaging of the ASLMB. **a, b.** Nyquist plot **a)** and the zoom-in plot **b)** of the ASLMB before the operando neutron imaging test. **c.** Voltage-capacity profiles of the ASLMB. **d, e.** Quantified neutron transmission b) and derivated transmission c) in the region along the cross section of the ASLMB. **f.** Voltage-time profiles of the ASLMB. **g.** Dynamic transmission evolution during charging and discharging processes. The green, warm, and cold colors represent no obvious changes, enriched Li, and Li depletion, respectively, compared with the pristine state in the charging process and with the fully charge state in the discharging process.

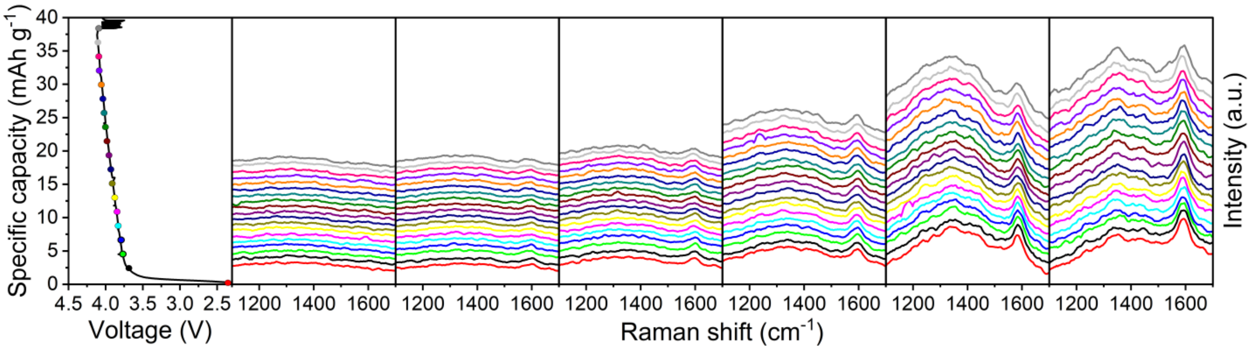

Figure S15. Raman spectra corresponding to the different state of charge.

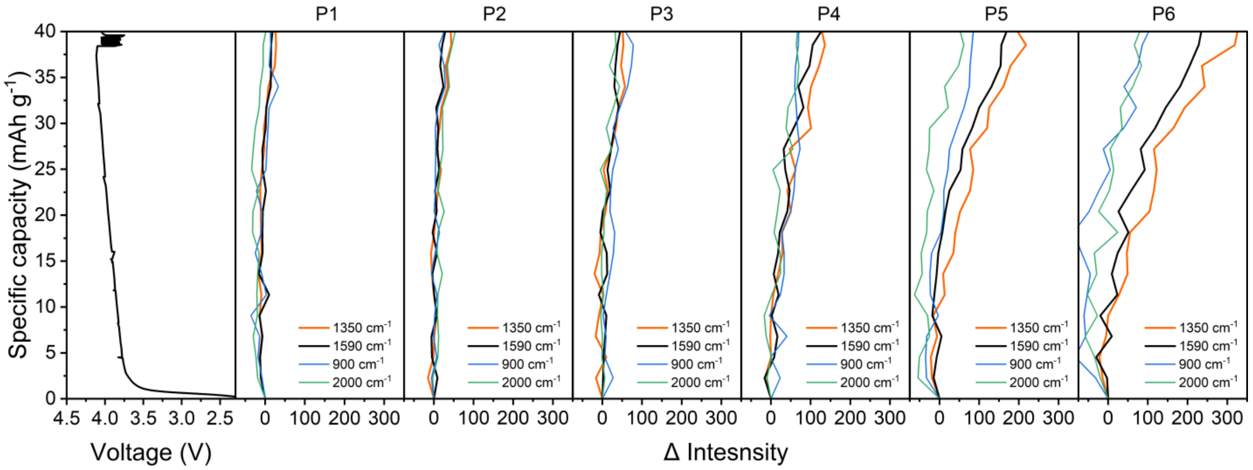

Fig. S16. The intensity enhancements at Raman shifts of 1350 cm^{-1}, 1590 cm^{-1}, 900 cm^{-1}, and 2000 cm^{-1} in different locations.

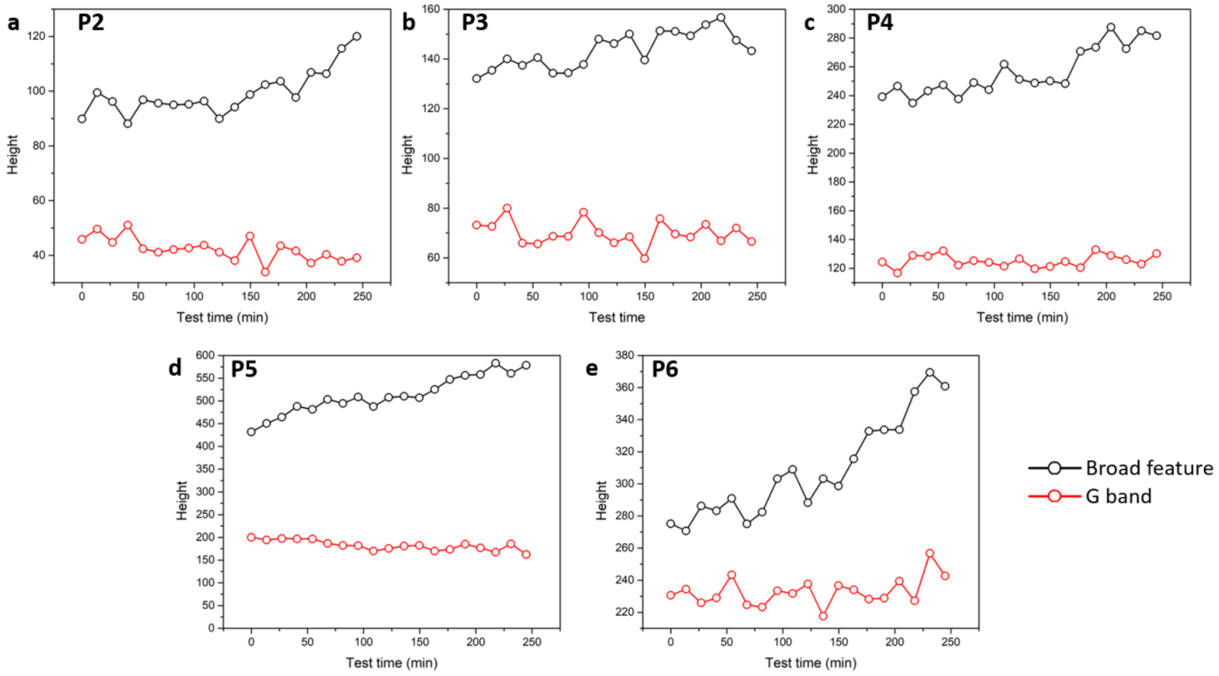

Fig. S17. The intensity evolutions of Peaks at 1350 cm^{-1} and 1590 cm^{-1} were fitted by Lorentzian during test.

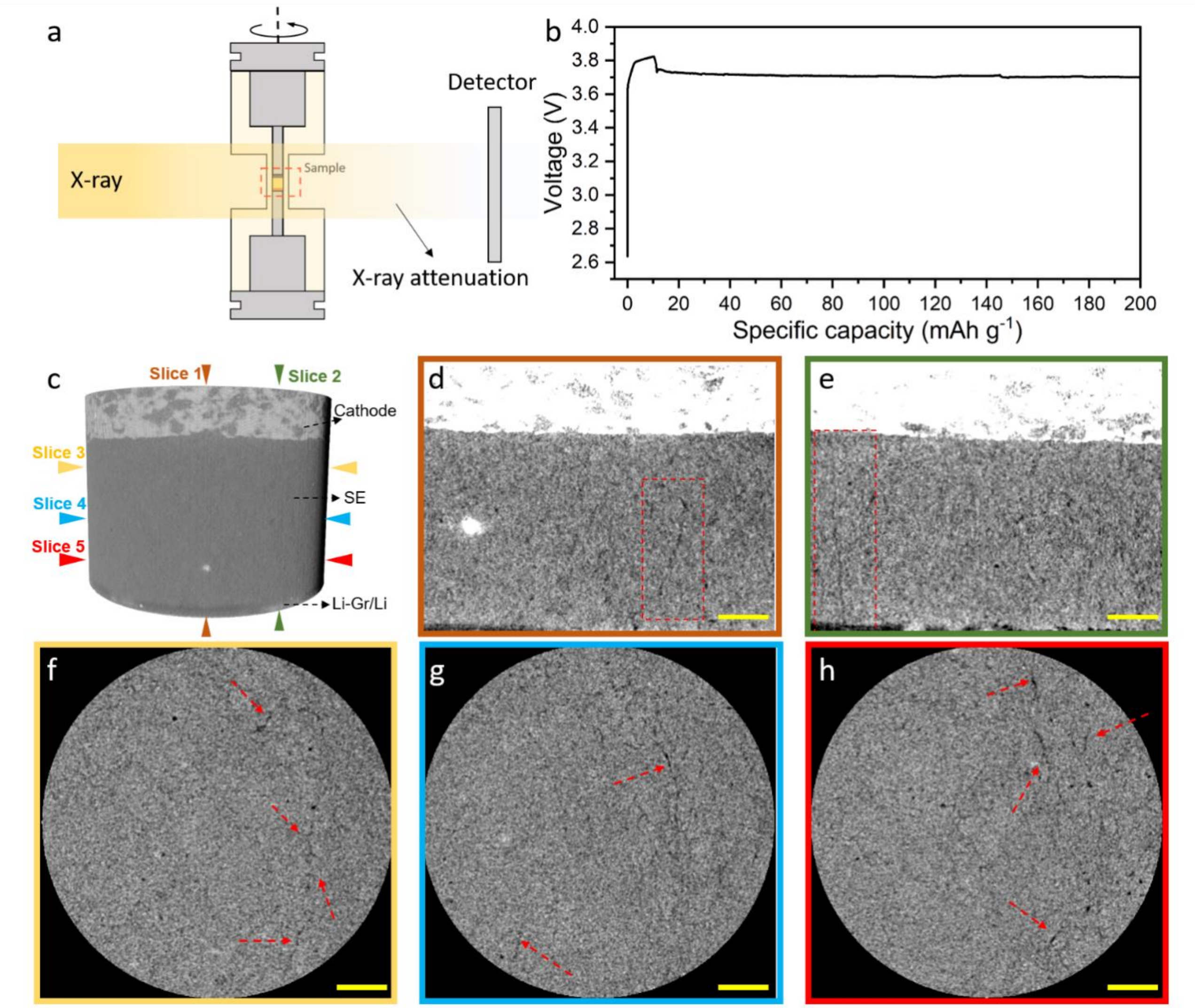

Fig. S18. Ex situ XCT investigation. **a,** Schematic of the XCT test process. **b,** Galvanostatic charge profile of the ASLMB in the XCT test. **c,** Reconstructed 3D structure of the ASLMB. The locations of five slices are labeled in the figure. **d-h,** Reconstructed 3D images of Slice 1-5. The dash boxes in b) and c) highlight the vertically grown cracks. The dash arrows in d), e), and f) show the horizontal cracks. The scale bar is 100 μm.

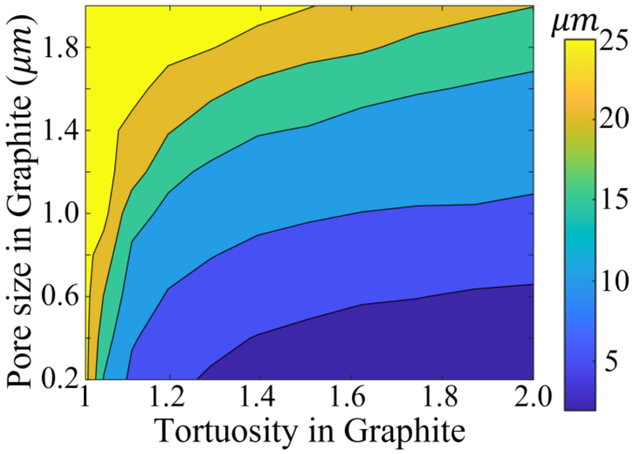

Fig. S19. Li extrusion at different graphite microstructure.

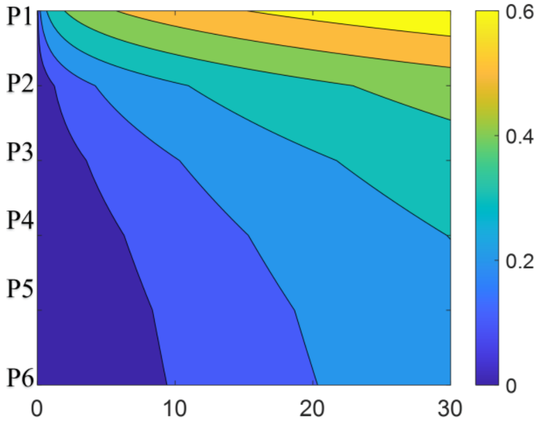

Fig. S20. Li content without stacking pressure.

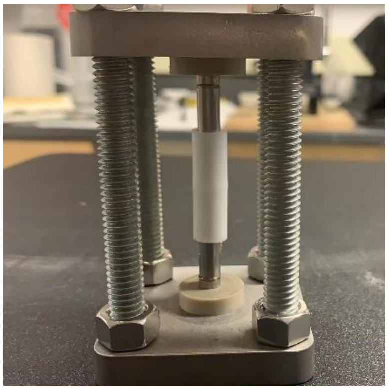

Fig. S21. Photo of the cell for operando neutron imaging investigation.

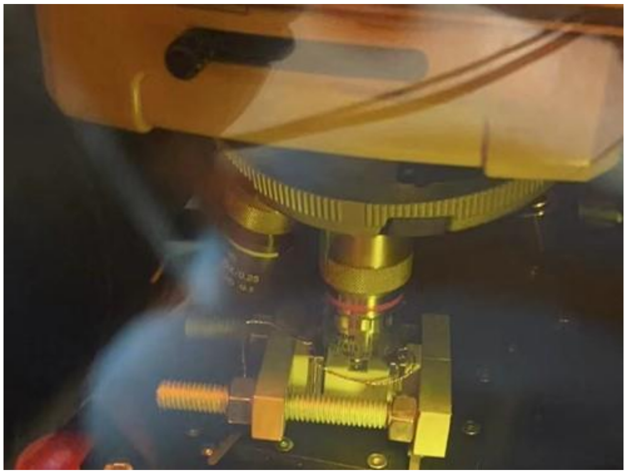

Fig. S22. Photo of the Raman inside the glovebox.

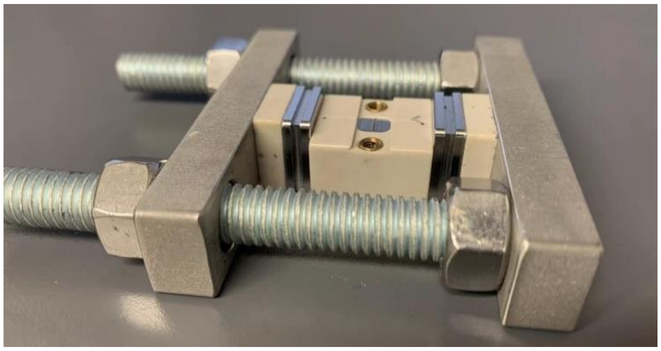

Fig. S23. Photo of the cell for operando Raman investigation

Supplemental Tables

Table S1. Broad feature and G peak information in Li-graphite and pristine graphite

	G peak position (cm^{-1})	G peak intensity (a.u.)	Broad feature position (cm^{-1})	Broad feature intensity (a.u.)	I_B/I_G
P1	1604.7	22.6	1337.5	72.2	3.42
P2	1598.3	45.8	1332.4	89.9	1.96
P3	1597.5	73.2	1329.9	132.1	1.81
P4	1595.9	124.5	1331.3	239.2	1.92
P5	1588.3	176.8	1352.0	331.6	1.88
P6	1593.1	230.6	1355.5	275.1	1.19
Pristine graphite	1583.6	96.2	1336.3	13.5	0.14

Table S2. Key parameters used in this computational modeling.

Name	Symbol	Unit	Value
Anodic/cathodic transfer coefficients	α_a, α_c	1	0.5
Exchange current density at Li-graphite and SE interface	i_{exc}	mA/cm^2	1.3
Ionic conductivity in the Li-Gr	σ_{Li^+}	mS/cm	[5~0.05]
Electronic conductivity in the Li-Gr	σ_{e^-}	mS/cm	10^4
Bulk modulus of the Li metal	K	GPa	11
Thickness of the Li-Gr	L	μm	26

References

1. Baddour-Hadjean, R.; Pereira-Ramos, J.-P., Raman Microspectrometry Applied to the Study of Electrode Materials for Lithium Batteries. *Chemical Reviews* **2010,** *110* (3), 1278-1319.
2. Ferrari, A. C.; Basko, D. M., Raman spectroscopy as a versatile tool for studying the properties of graphene. *Nature Nanotechnology* **2013,** *8* (4), 235-246.
3. Gray, M. J.; Kumar, N.; O'Connor, R.; Hoek, M.; Sheridan, E.; Doyle, M. C.; Romanelli, M. L.; Osterhoudt, G. B.; Wang, Y.; Plisson, V.; Lei, S.; Zhong, R.; Rachmilowitz, B.; Zhao, H.; Kitadai, H.; Shepard, S.; Schoop, L. M.; Gu, G. D.; Zeljkovic, I.; Ling, X.; Burch, K. S., A cleanroom in a glovebox. *Review of Scientific Instruments* **2020,** *91* (7), 073909.
4. Porz, L.; Swamy, T.; Sheldon, B. W.; Rettenwander, D.; Frömling, T.; Thaman, H. L.; Berendts, S.; Uecker, R.; Carter, W. C.; Chiang, Y.-M., Mechanism of Lithium Metal Penetration through Inorganic Solid Electrolytes. *Advanced Energy Materials* **2017,** *7* (20), 1701003.
5. Ning, Z.; Jolly, D. S.; Li, G.; De Meyere, R.; Pu, S. D.; Chen, Y.; Kasemchainan, J.; Ihli, J.; Gong, C.; Liu, B.; Melvin, D. L. R.; Bonnin, A.; Magdysyuk, O.; Adamson, P.; Hartley, G. O.; Monroe, C. W.; Marrow, T. J.; Bruce, P. G., Visualizing plating-induced cracking in lithium-anode solid-electrolyte cells. *Nature Materials* **2021**.

6. Mayur, M.; DeCaluwe, S. C.; Kee, B. L.; Bessler, W. G., Modeling and simulation of the thermodynamics of lithium-ion battery intercalation materials in the open-source software Cantera. *Electrochimica Acta* **2019,** *323*, 134797.

7. Barroso-Luque, L.; Tu, Q.; Ceder, G., An Analysis of Solid-State Electrodeposition-Induced Metal Plastic Flow and Predictions of Stress States in Solid Ionic Conductor Defects. *Journal of The Electrochemical Society* **2020,** *167* (2), 020534.

8. Schmitter, E. D. In *Modelling massive forming processes with thermally coupled fluid dynamics*, Proceedings of the COMSOL Multiphysics User's Conference, 2005.

9. Perzyna, P., Fundamental Problems in Viscoplasticity. In *Advances in Applied Mechanics*, Chernyi, G. G.; Dryden, H. L.; Germain, P.; Howarth, L.; Olszak, W.; Prager, W.; Probstein, R. F.; Ziegler, H., Eds. Elsevier: 1966; Vol. 9, pp 243-377.

10. LePage, W. S.; Chen, Y.; Kazyak, E.; Chen, K.-H.; Sanchez, A. J.; Poli, A.; Arruda, E. M.; Thouless, M. D.; Dasgupta, N. P., Lithium Mechanics: Roles of Strain Rate and Temperature and Implications for Lithium Metal Batteries. *Journal of The Electrochemical Society* **2019,** *166* (2), A89-A97.

11. Wilkins, A.; Green, C. P.; Ennis-King, J., An open-source multiphysics simulation code for coupled problems in porous media. *Computers & Geosciences* **2021,** *154*, 104820.
